# Hazards and Control Measures among Artisanal and Small-Scale Gold Miners in Zimbabwe

**DOI:** 10.5334/aogh.3621

**Published:** 2022-03-15

**Authors:** Josephine Singo, John Bosco Isunju, Dingani Moyo, Nadine Steckling-Muschack, Stephan Bose-O’Reilly, Antony Mamuse

**Affiliations:** 1 Centre for International Health, University Hospital, LMU Munich, Leopoldstrasse 5, D-80802 Munich, Germany; 2 Devsol Consulting, P. O. Box 73201 Clock Tower Kampala, Uganda; 3 Exceed Institute of Safety Management and Technology, Plot 595, Block 9, Kweesa Road, Lubaga Kampala, Uganda; 4 Disease Control and Environmental Health Department, Makerere University School of Public Health P.O.Box 7072 Kampala, Uganda; 5 School of Public Health, University of the Witwatersrand, Johannesburg, South Africa; 6 Faculty of Medicine, National University of Science and Technology, Bulawayo, Zimbabwe; 7 Faculty of Medicine, Midlands State University, Private Bag 9055, Senga Road, Gweru, Zimbabwe; 8 Institute and Clinic for Occupational, Social, and Environmental Medicine, University Hospital, LMU Munich, Ziemssenstr. 5, D-80336, Munich, Germany; 9 Institute of Public Health, Medical Decision Making and Health Technology Assessment, Department of Public Health, Health Services Research and Health Technology Assessment, UMIT (Private University for Health Sciences, Medical Informatics, and Technology), Eduard-Wallnoefer-Centre 1, A-6060 Hall in Tirol, Austria; 10 Klinikum Osnabrueck GmbH, Am Finkenhuegel 1, D-49076 Osnabrueck, Germany; 11 Department of Geosciences, Faculty of Engineering & Geosciences, Midlands State University, Private Bag 9055, Senga Road, Gweru, Zimbabwe

## Abstract

**Background:**

In 2017 around 14–19 million miners were exposed to multiple hazards in artisanal and small-scale gold mining (ASGM). ASGM is characterized by basic and compromised mining methods with either very limited control of hazards or none at all. There is little knowledge about health and safety among artisanal and small-scale gold miners in Zimbabwe.

**Objective:**

This study explores the interaction between hazards, control measures, and health and safety in Zimbabwe’s ASGM.

**Methods:**

Triangulation and mixed methods were applied using standardized questionnaires, Hazard Identification and Risk Assessment (HIRA), focus group discussions (FGDs), and summary notes from in-depth interviews (IDIs). Data were analyzed using descriptive statistics, regression analysis, and thematic analysis.

**Findings:**

Quantitative data were collected through HIRA, which was conducted on 34 mining sites. 401 participants, selected through multi-stage sampling, were assessed through standardized questionnaires. Qualitative data was collected through six FGDs, and existing summary notes from 84 IDIs. The most prioritized hazards from the questionnaires were silica dust, noise, and workplace violence as indicated by 238 (62.0%), 107 (26.8%), and 104 (26.7%) respondents (respectively). HIRA identified noise, dust, unsafe shafts, violence, poor sanitation, and poor hygiene as key hazards requiring urgent attention. A key finding of this study was the poor application of the hierarchy of controls in managing workplace hazards. After adjusting for confounders, association with experiencing health and safety challenges was working underground (AOR = 2.0, p = 0.03), workplace violence (AOR = 3.3, p = 0.002), and long working hours (AOR = 2.8, p = 0.019). Injuries and fatalities were common without mitigation strategies.

**Conclusions:**

ASGM in Zimbabwe is characterized by underground mining, long working hours, and workplace violence. The poor application of the hierarchy of controls is characterized by increased workplace injuries and fatalities. We recommend following the hierarchy of control measures in ASGM: elimination, substitution, engineering, administrative, and personal protective equipment.

## Introduction

Artisanal and small-scale gold mining (ASGM) involves gold mining through basic rudimentary and semi-mechanized methods [1]. ASGM is usually associated with poor health and safety standards [1]. In 2017, an estimated 14–19 million miners were employed in ASGM globally [2]. In Zimbabwe, over 500,000 people are involved in artisanal and small-scale mining, which is largely ASGM [3]. ASGM contributes directly to the sustenance of at least one million in Zimbabwe [4]. Mining regulations are the same for ASGM and large mines in Zimbabwe [5], and there are plans to formalize ASGM [6].

**Figure 1:**
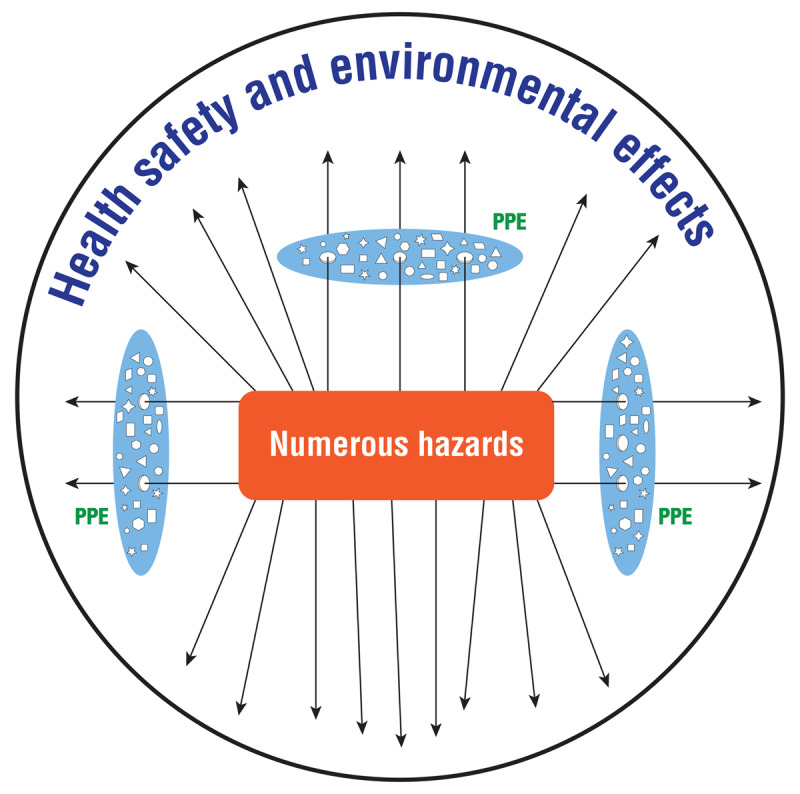
Numerous hazards and compromised control measures in ASGM: Interaction between hazards, PPE use and health, safety and environmental effects in ASGM in Zimbabwe.

Mining is a high-risk sector requiring effective control measures to protect workers’ health [5,7,8]. Occupational health and safety aims to prevent, manage, and control occupational hazards [9]. The hierarchy of control measures, from the most to the least effective, includes elimination, substitution, engineering, administrative, and personal protective equipment (PPE) [10]. Elimination and substitution are more feasible at the design and development stages and more challenging for existing operations [10]. Engineering control measures are designed to address hazards at the source [11] and are effective and less independent of human behavior, despite the cost [10]. PPE is less effective, owing to dependence on human effort [10,11]. Mitigation control measures involves interventions to prevent and manage health and safety incidences [11]. The Swiss cheese model assumes successive layers of control measures marked by “eyes,” of different shapes and sizes, which represent multiple weaknesses in those control measures and which are associated with accident opportunities [12]. This study assumes that the Swiss cheese model could be applicable to ASGM, which is associated with hazardous working conditions [1,8,13] and compromised control measures. ***Figure 1*** illustrates the interaction between hazards, PPE use, and health, safety, and environmental effects in ASGM.

The figure assumes that PPE is the most common control measure in ASGM in Zimbabwe. While the Swiss cheese model displays various successive layers of control measures [12], ***Figure 1*** assumes one layer of PPE characterized by various weaknesses and missing layers of control measures, resulting in increased adverse health safety and environmental effects in ASGM.

Global studies on ASGM found hazardous working conditions including dust, chemicals, and noise, as well as ergonomic, psycho-social, environmental, and biological hazards [14,15,16]. Studies on ASGM in Kadoma noted unsafe mining pits, lack of PPE use, lack of safe drinking water and toilets, low hygiene, and poor waste management [16,17]. These conditions were associated with accidents, acute respiratory infections, tuberculosis, malaria, and sexually transmitted infections [16,17]. It was also found that ASG miners have limited to near-absent access to health care [16,17]. A cross-sectional survey among ASG miners in Midlands and Matabeleland South Provinces in Zimbabwe reported a high prevalence of silicosis (11.2%), tuberculosis (TB) (4.0%), and HIV (23.5%) [18]. A review on occupational health and safety (OHS) in Southern Africa revealed that OHS is grossly inadequate in the informal sector [19], which was confirmed by a global review on OHS in ASGM that found no studies on comprehensive occupational health interventions in ASGM [20]. ASGM informally employs more workers than large mines in many countries [8], without basic safety standards [8].

This study explores the interaction between hazards and control measures in ASGM in Zimbabwe. The objectives of this study are:

1. identifying hazards and controls in ASGM in Zimbabwe,
2. exploring the interaction between hazards and control measures, and
3. assessing the associated health safety and environmental effects.

## Methods

The study was conducted from November to December 2020 in Shurugwi and Kadoma, Midlands and Mashonaland West provinces of Zimbabwe that are characterized by high ASGM activities [21]. Triangulation, which involved integration of different methods to tackle the research phenomenon from different angles and the mixed method design that complements quantitative and qualitative methods on the same research problem were used for a comprehensive study. Data were collected using standardized questionnaires [22], Hazard Identification and Risk Assessment (HIRA), focus group discussions (FGDs), and summary notes from qualitative interviews taken from another survey that was conducted on ASGM in Kadoma in 2017 [17]. HIRA, standardized questionnaires, and focus group discussions were conducted in 2020 to both quantify and understand hazards, controls, and experienced health and safety effects [22]. All data collection tools were available in Shona and Ndebele. Data collection tools were pre-tested among eight miners from two mining sites in Kadoma and Gwanda in Zimbabwe. The tools were modified and translated into Ndebele and Shona by experienced translators and approved, together with the study protocol, by the Medical Research Council of Zimbabwe. All participants gave written informed consent to participate in the study.

## Quantitative Methods

### Questionnaires

Miners were interviewed using standardized questionnaires. The target population was ASG miners working in Kadoma and Shurugwi. The study sample was selected by multi-stage sampling. Firstly, active mining areas involved in rudimentary and more mechanized mining methods were selected purposively in Kadoma and Shurugwi. Secondly, stratified simple random sampling was conducted through reshuffling names of identified active sites practicing rudimentary and more mechanized mining methods. Lastly, participants in the selected sites were randomly selected by means of tossing a coin (considering proportional gender inclusion).

The questionnaires (Appendix 1), which was administered by the interviewer, focused on health and safety issues and was designed based on previous ASGM surveys in Kenya and Zimbabwe [17,23]. Consenting adults aged eighteen years and above who had been involved in ASGM for at least six months were included. Drunk, disinterested, and non-cooperative respondents were excluded. Sites which were practicing rudimentary and more mechanized mining were visited in eight mining areas in Kadoma and Shurugwi.

### Hazard Identification and Risk Assessment (HIRA)

The modified, standardized, and tested University of Cape Town baseline HIRA tool (Appendix 2) [24], with a risk matrix of 1 to 5; likely, minor, moderate, high, very high (Appendix 3) [25]. The assessment was conducted by a team trained through presentations that involved was used active participation. Training was conducted by the principal investigator experienced in risk assessment, with input from ASGM association leader experienced in training and researches in ASGM. The HIRA tool was fully developed and tested by other researchers (Appendix 3). Probability was weighted against the severity of impacts and frequency. The weighted risk (%) was calculated using the formula [24]:

Risk = \frac{{\left[ {Probability \times Severity\left({I + P + E + C} \right) \times Frequency} \right] \times 100}}{{500}}

Key (for the formula)

I Injury/disease

P Production

E Environment

C Cost

HIRA was conducted by the research team through direct observation, walk-through surveys, and informal discussions with workers on thirty-four sites in eight mining areas in Kadoma and Shurugwi. The final risk assessment score was moderated with input from experienced miners and ASGM Association leader who had more than thirty years in ASGM, was verified and reviewed by mining engineer from the Ministry of Mines and Mining Development, and independent ASGM experts, experienced in mining safety and ASGM in Zimbabwe. Risk scores were profiled for priority of action as shown below.

**Table:** 


A	75%–100%	immediate attention needed

B	60%–74%	attention required in 1 week

C	45%–59%	attention required in 1 month

D	30%–44%	attention needed in 6 months

E	15%–29%	attention required in 12 months

F	1%–14%	attention required as soon as possible [24]


Risk profiling determined the urgency of response measures.

## Qualitative Methods

### Focus Group Discussions (FGDs)

FGD participants who were miners for at least six months were selected by a local association leader experienced in ASGM through snowballing, which was guided by references from initial known contacts from different mining sites. A FGD guide (Appendix 4) was used to explore the following themes: hazards, existing controls, challenges on health and safety, and associated effects. The focus group discussions were conducted in Shona and were transcribed into MS Word in Shona before they were translated and transcribed into MS Word in English by experienced translators (and then verified by them). Three FGDs were conducted in the dry season in 2017 in Kadoma during a different survey [17], while three FGDs were conducted in the rainy season in Shurugwi in 2020. Data from different seasons were used to understand occurrences of hazards in different seasons.

### Existing Summary Notes from In-Depth Interviews

Summary notes from qualitative interviews taken in an earlier survey [17] that was conducted by University Hospital, LMU Munich, in October 2017 were used for the study. Snowball sampling was used through initial contact with potential participants. Nine mining sites were visited during the 2017 survey [17]. The in-depth interviews were conducted by a University Hospital, LMU Munich, research team. Themes for IDI included experiences with hazards and risks and PPE use (Appendix 5). The in-depth interviews were conducted in Shona, summarized, and translated into English.

### Data Analysis

Data from the questionnaires and HIRA tool were entered, cleaned, and analyzed in SPSS version 20 (IBM SPSS, Chicago, Illinois, USA). HIRA risk scores were weighted, calculated into percentages, and categorized from A to F [24]. For risk assessment, the mode, median, and quartiles were calculated. Categorical data from the questionnaires were summarized using frequencies and percentages. The association between experiencing health and safety challenges and predictor variables such as age, gender, district, working hours, and violence was assessed using binary logistic regression and was presented as odds ratios (ORs) and adjusted odds ratios (AORs). The level of significance was set at p = <0.05. Qualitative data from FGDs and IDIs were analyzed using thematic analysis to explore the various hazards experienced by the miners, experienced health and safety effects, and experiences with accessing health services. Quotations from FGDs and notes from in-depth interviews were used to explain the findings from the questionnaires. As the standard questionnaires and HIRA had comprehensive results sufficient for this paper, coding and further analyses of data from FGDs and IDIs were not conducted for this paper.

## Ethical Approval

The study was approved by the University of Munich Ethics Committee (Project 20-068) and the Medical Research Council of Zimbabwe (MRCZ/A/2603). Consent was sought from local authorities, mine owners, and all participants. There was voluntary participation and signing of informed consent prior to data collection. Questionnaires were numbered without names to ensure confidentiality. The survey conducted in Kadoma in 2017 in the dry season was approved by MRCZ (MRCZ/B/1425) [17] and the University of Munich Ethics Committee (17-665) [17].

## Results

Quantitative data from the questionnaires had 401 respondents, with a response rate of 88%. Thirty-four site assessments were carried out during the study. Six FGDs were conducted. Summary notes from a different study with 84 respondents (15 women and 69 men) were also used [17].

### Socio-Demographic Characteristics of the Questionnaires Study Population

The total sample consisted of 220 respondents from Kadoma and 181 from Shurugwi. The study population consisted of 69 women and 332 men. Fifty-one percent of the study population was married. Two hundred and twenty respondents were within the 18–35 age category as indicated in ***Table 1***.

**Table 1:** Socio-demographics: Socio-Demographic Characteristics of the Miners from Kadoma and Shurugwi in Zimbabwe in 2020 (n = 401).


CHARACTERISTICS		N (%)	TOTAL

**Population per district**		401 (100)	401

**District n (%)**	Kadoma	220 (54.9)	

**Mine category (n (%)**	Shurugwi	181 (45.1)	401

	Rudimentary	78 (19.5)	

**Gender n (%)**	More mechanized	323 (80.5)	401

	Female	69 (17.2)	

**Marital status n (%)**	Male	332 (82.8)	401

	Single	126 (31.9)	

**Age (n (%)**	Married	202 (51.1)	

	Separated	17 (4.3)	

	Divorced	28 (7.1)	

	Widowed	22 (5.6)	395

	18–35 years	212 (56.1)	

**Education level n (%)**	36–50 years	130 (34.4)	

	Above 50 years	36 (9.5)	378

	No formal school	28 (7.1)	

**Monthly Earnings n (%)**	Primary	59 (14.9)	

	Secondary	241 (60.9)	

	Tertiary	39 (9.8)	

	Vocational	29 (7.3)	396

	No Earnings	7 (1.9)	

**Roles n (% of cases)**	Less than 100 US$	212 (56.7)	

	Above 100–500 US$	13 (34.8)	

	Above 500–1 000 US$	24 (6.4)	

	Above 1000 US$	1 (0.3)	374

	Digging	211 (65.3)	

**Daily working hours n (%)**	Moving ore manually	59 (18.3)	

	Blasting	51 (15.8)	

	Loading	44 (13.6)	

	Washing/panning	33 (10)	

	Cooking	26 (7.9)	

	Amalgam burning	24 (7.3)	

	Milling	24 (7.3)	

	Sponsoring	22 (6.8)	

	Supervision	22 (6.8)	

	Mine owner	19 (5.9)	

	Gold buying	14 (4.3)	549 **(Total cases)**

	1–8 hours	259 (66.9)	

**Working underground n (%)**	Above 8–16 hours	82 (21.2)	

	Above 16–24 hours	46 (11.9)	387

	Working underground yes	201(52.3)	385

**Working arrangements n (%)**	Shares	229 (61.2)	

	Salary	89 (23.8)	

	Contract	35 (9.4)	

	Individual	21 (5.6)	374


Twelve percent of the participants worked for 17 to 24 hours daily, and 52% worked underground. More than 50% of the participants earned less than 100 United States Dollars (US$) per month. In addition, FGDs revealed challenges with unreliability of earnings, as one miner expressed,

*‘The challenge here is that people do not get money on time. The gold doesn’t always come. You can work for 2–3 months without getting money while working. You can be affected working like this when nothing is coming out. We need to get money to live well*.’ – Male miner, FGD^1^

## Priority Hazards Perceived by Miners

The most prioritized hazard was silica dust, as indicated by 238 (62%) of the respondents, while noise and workplace violence were indicated by 107 (26.8%) and 104 (26.7%) respondents, respectively. Workplace violence was associated with loss of ore, gold, and equipment (n = 48, 53.9%) and loss of shafts (n = 16, 18%), as was confirmed by qualitative data (***Table 5***). Noise was common on sites that had equipment. For instance, one site had excess noise from six stamp mills that were operating simultaneously. The workers at that site had helmets, work suits, and foot protection, but they had no ear protection and no gloves. The noise levels were too high for ear protection alone. As a result, hearing problems were also reported as presented further.

## Reported Standard Operating Procedures (SOPs) and Personal Protective Equipment (PPE) Use

Ninety-two respondents (25.6%) reported that they had SOPs, while 231 (59.8%) reported use of PPE, as shown below in ***Table 2***.

**Table 2:** Standard operating procedures (SOPs) and personal protective equipment (PPE) use: SOPs and PPE use reported by miners in Kadoma and Shurugwi, Zimbabwe, in 2020.


STANDARD OPERATING PROCEDURES AND PPE USE		N (%)	TOTAL

**Standard Operating Procedures n (%)**		92 (25.6)	360

**Use of PPE n (%)**		231 (59.8)	386

**Replacement of PPE n (%)**	Frequently	37 (34.9)	

**Means of getting PPE n (%)**	Rarely	49 (46.2)	

	Never	20 (18.9)	295

	Provided at work	104 (46.4)	

**Reasons for non-PPE use n (% of cases)**	Bought for self	104 (46.4)	

	Co-worker	13 (5.8)	

	Friend or family	11 (4.9)	

	Not Provided	68 (29.7)	106

	I don’t know	68 (29.7)	

	Lack of awareness	39 (17)	

	Not affordable	35 (15.3)	

	Not comfortable	13 (5.7)	

	Not necessary	11 (4.8)	233 **(Total cases)**


Six percent of the participants were mine owners (***Table 1***). PPE was mainly provided at work or self-sourced. In Shurugwi, it was common for miners to source their own PPE. In Kadoma, some mine owners and sponsors were providing PPE to complement the PPEs sourced by individual miners. However, observed PPE use was lower than reported (***Tables 4*** and ***5***). Replenishing worn-out PPE was a challenge for more than fifty percent of the respondents. Thirteen of the respondents indicated that non-compliance to PPE use was due to discomfort associated with PPE use, which could stem from negative perceptions. The migratory nature of ASGM also posed an obstacle to PPE use (***Table 5***). Furthermore, the use of PPE alone was not sufficiently protective against mine collapses and heavy rock falls. As one respondent stated,

*“Where they work is unsafe. They have PPE. They use it. PPE cannot stop mine collapses. Gumboots cannot prevent a heavy stone. PPE cannot protect a person against a heavy rock fall.”* -Female miner, FGD^2^

The association between experiencing health and safety challenges and exposure to hazards is shown in ***Table 3***.

**Table 3:** Exposure to hazards and health and safety challenges: Association between experiencing health and safety issues and exposure to hazards reported by miners in Kadoma and Shurugwi, Zimbabwe, during the 2020 rainy season.


CHARACTERISTIC	TOTAL	HEALTH & SAFETY CHALLENGES		OR (95% CI)	AOR (95% CI)	
	
		Number	(%)†			P VALUE

**Overall**	393	178	(45)			

**Gender (n = 393)**						

Male	326	148	(45)	1.0 (0.6–1.7)	0.5 (0.2–1.4)	0.1

Female	67	30	(45)	Reference	Reference	

**Age (n = 370)**						

>50	36	15	(42)	Reference	Reference	

36–50	128	59	(46)	1.7 (0.5–1.7)	1.1 (0.4–3.3)	0.9

18–35	206	90	(44)	1.3 (0.5–2.2)	1.3 (0.5–3.3)	0.7

**District (n =393)**						

Kadoma	215	94	(44)	Reference	Reference	

Shurugwi	178	84	(47)	1.2 (0.8–1.7)	1.1 (0.6–2)	0.7

**Working underground n = (376)**						

Yes	197	100	(51)	1.6 (1.1–2.4)**	2.0 (1.1–5.0)**	0.03**

No	179	70	(39)	Reference	Reference	

**Moving up and down the shaft (n = 379)**						

Yes	43	43	(100)	2.5 (2.5–3.3)**	–	0.1

No	336	122	36)	Reference	Reference	

**Crushing (n = 379)**						

Yes	22	22	(100)	2.5 (2.0–2.5)*	–	0.1

No	357	143	(40)	Reference	Reference	

**Opening shaft (n = 379)**						

Yes	21	21	(100)	2.5 (2–2.5)**	–	0.1

No	358	144	40)	Reference	Reference	

**Workplace violence (n = 382)**						

Yes	102	68	(67)	3.4 (2.1–5.5) **	3.3 (1.4–5.0)**	0.002**

No	280	103	(37)	Reference	Reference	

**Working hours (n = 379)**						

17–24	45	31	(69)	2.5 (1.1–5.0)**	2.8 (1.2–6.5)	0.019 **

9–16	80	38	(48)	3.3 (1.7–5.0)	1.4 (0.6–1.4)	0.4

1–8	254	106	(42)	Reference	Reference	


AOR = Adjusted Odds Ratio; CI = 2-sided confidence interval; † = row percentages; ** = statistically significant.

There were no significant differences in odds of experiencing health and safety challenges by gender, district, and age. After adjusting for other variables in the model, the odds of experiencing health and safety challenges were higher for miners who reported working underground, AOR= 2.0, [1.1–5.0], miners who had experienced workplace violence, AOR= 3.3 [CI= 1.4–5.0], and miners who had long daily working schedules of 17–24 hours, AOR= 2.8[1,2–6.5]. A case of three miners who were trapped underground around three o’clock in the morning in Zvishavane during the 2020 rainy season was mentioned. Such cases were reported as typical (***Table 5***).

One hundred and seventy-eight participants (45%) reported having experienced health and safety challenges (***Table 3***). The major health and safety problems experienced were respiratory (n = 33, 26.6%), musculoskeletal (n = 29, 23.4%), stress (n = 28, 22.6%), and injuries (n = 24, 19.4%). Hearing (n = 10, 8.1%), and reproductive problems (n = 4, 3.2%) were also reported. Experienced health and safety problems could be linked to exposure to dust, heavy lifting, and unsafe shafts (illustrated in ***Table 3***). FGDs revealed that stress was related to sicknesses among ASG miners (***Table 5***). Stress was also resulting from low and erratic wages, as presented above. The common reported injuries were fractures (n = 34, 52.2%), cuts (n = 24, 41.4%), and bruises (n = 22, 37.9%). The reported ever-injured body parts were hand(s) (n = 38, 28.1%), leg(s) (n = 32, 23.7%), finger(s) (n = 24, 17.8%), head (n = 19, 14.1%) and chest (n = 15, 11.1%). One miner, in FGD, explained that loss of fingers was common at the milling centers (***Table 5***). Protection of miners at milling centers was compromised as explained above.

Access to health care was limited. Consequently, there was a tendency of seeking health care from alternative options other than mainstream care. Alternative health-seeking options included prophet/prayers (n = 26, 57.8%), pharmacy (n = 9, 20%), traditional healers/herbalist (n = 3, 6.7%), self-medication (n = 2, 4.4%) and illegal drug dealers (n = 2, 4.4%). Miners resorted to alternative health-seeking options because they could not afford hospital costs, as further revealed in ***Table 5***.

## Identified Hazards and Risks (HIRA)

The identified hazards and risk scores are illustrated below.

The majority of the identified hazards required immediate attention. Silica dust and noise required immediate action on 16 (55%) and 10 (43%) individual sites, respectively. Observed sources of silica dust were dumps, dry blasting, lashing, and dry crushing. Engineering controls such as water sprays for blasting, crushing, and milling (hammer mills) were lacking on visited sites. Furthermore, risk assessment was conducted during the rainy season, when the majority of the miners had no protection against dust—unlike during the dry season in 2017, when more miners had cloths, such as mutton cloths, for protection against high levels of dust. PPE use had challenges as confirmed in ***Tables 2*** and ***5***. Occupational alcohol abuse and smoking were also reported in ***Table 5***. Unsafe shaft support was associated with fatalities (***Table 5***). ***Table 4*** shows uncovered mining pits, and stagnant water which is associated with mosquitoes and malaria. Miners also reported injuries, respiratory problems, and musculoskeletal problems, which could be linked to exposure to unsafe shafts, dust, and heavy lifting in ***Table 4***, as mentioned above.

**Table 4:** Hazards and risks in ASGM: Identified hazards and weighted risk scores (%) from mining sites in Kadoma and Shurugwi, Zimbabwe, during the 2020 rainy season (n = 34).


	SITES (N)	SITES IN CATEGORY A 75–100% N (%)	MODE	MEDIAN	QUARTILES 25^TH^	50TH	75^TH^

**Environmental and physical hazards**							

Noise	23	10(43)	34(D)	55(C)	34(D)	55(C)	80(A)

Uncovered old mining pits	21	5(24)	40(D)	58(C)	42(D)	58(C)	75(A)

Stagnant water	8	1(13)	22(E)	27(E)	22(E)	27(E)	52(C)

Lack of toilets	24	8(33)	100(A)	37(D)	30(D)	37(D)	79(A)

Mine contaminated drinking water	7	4(57)	I00(A)	100(A)	22(E)	100(A)	100(A)

Indecent shelter	7	1(14)	5(F)	47(C)	22(E)	47(C)	61(B)

Water pools in panning sites	1	1(100)	100(A)	100(A)	100(A)	100(A)	100(A)

Mining activities around homesteads	15	6(40)	100(A)	64(B)	30(D)	64(B)	81(A)

Electricity	6	6(100)	100(A)	98(A)	84(A)	98(A)	100(A)

Clutter	8	3(36)	100(A)	46(C)	25(E)	46(C)	94(A)

Lack of fencing/signage	24	9(38)	100(A)	61(B)	30(D)	83(A)	100(A)

Lack of PPE	25	12(48)	100(A)	70(B)	37(D)	70 (B)	94(A)

**Mechanical hazards**							

Unsafe shaft support	18	9(50)	100(A)	83(A)	51(C)	83(A)	100(A)

Equipment	19	8(42)	68(B)	68(B)	52(C)	68 (B)	90(A)

**Chemical hazards**							

Chemicals	25	17(68)	100(A)	100(A)	66(B)	100(A)	100(A)

Contamination of food	6	1(17)	32(D)	32(D)	24(E)	32(D)	72(B)

Mine contaminated drinking water	7	4(57)	I00(A)	100(A)	22(E)	100(A)	100(A)

Chemical contamination of farmland	7	4(57)	32(D)	80(A)	32(D)	80(A)	100(A)

Mine waste	20	4(20)	22(E)	43(D)	35(D)	43(D)	68(B)

Silica dust	29	16(55)	65(B)	75(A)	65(B)	75(A)	86(A)

**Ergonomic hazard(s)**							

Confined working space	21	3(14)	24(E)	32(D)	22(E)	32(D)	72(B)

Manual Lifting	21	7(33)	54(C)	62(B)	38(D)	62(B)	77(A)

**Psycho-social hazards**							

Conflicts & violence	5	1(20)	32(D)	40(D)	34(D)	40(D)	68(B)

Child labor	21	5(24)	100(A)	48(C)	35(D)	48(C)	75(A)

Alcohol abuse & smoking	11	7(64)	100(A)	100(A)	24(E)	100(A)	100(A)

Prostitution	6	4(67)	100(A)	90(A)	71(B)	90(A)	100(A)

**Security hazards**							

Lack of security guards on-site	4	1(25)	22(E)	58(C)	29(E)	58(C)	78(A)

**Biological hazards**							

Crocodiles & snake bites (gold panning)	5	–	22(E)	43(D)	29(E)	43(D)	55 (C)


**Table 5:** Hazards, health and safety effects; and accessibility of healthcare: Reported miners’ experiences with hazards, health safety, environmental effects, and availability of health services from FGDs and IDIs conducted among miners in Kadoma and Shurugwi in the 2017 dry season, and the 2020 rainy season.


THEMES	EXAMPLES OF QUOTES FROM IN-DEPTH INTERVIEWS (IDIS) AND FOCUS GROUP DISCUSSIONS (FGDS)

Physical hazards	*“Mining of pillars”* – Male miner, 31 years old, IDI *“noise from blasting and drilling from jackhammer without earmuffs” –* Male miner, 70 years old, IDI

	*“Heat and limited working space underground” –* Male miner, 48 years old, IDI

	*“Most of the shafts have poor ventilation below the expected standards”* – Male mine owner, FGD

	*“Shaking during blasting causes cracks, falling rocks and collapsing mines” –* Male miner, 25 years old, IDI

	*“Rock falls, collapsing mines, breaking ropes. People can get injured or die” –* Male miner, 48 years old, IDI

	*“Incidences of mining in small holes where people get closed in, in the rain season, and most of such cases in informal mining are realized later” –* Male miner, 70 years old, IDI

Chemical hazards	*“… dust especially from using the jackhammer” –* Miner, FGD *“Water for washing and drinking. In old shafts, the water can be contaminated with chemicals and acids from blasting fumes and acids” –* Miner, FGD

	*“The issue is thirst has no timetable. One can get thirsty at any time, especially when one is working. So when you are working and dehydrated, you do not think of health issues; you think of quenching the thirst and going back to work. So when we see clear water and do not get immediate effects after drinking, we assume all is well. So when working underground, we drink the mine water underground”* -Miner, FGD

Lack of PPE	*“Respirators are needed because those fumes from blasting can cause problems like TB. When the fumes are still there, there is a need to wear respirators. Respirators are needed; they must not run out. We are not using respirators because of a lack” –* Miner, FGD *“PPE wears out before one gets money for a replacement, and it is difficult to buy for oneself” –* Male miner, 26 years old, IDI *“We operate on share ownership. I have the capital to sponsor the mine, but both the mine owner and the miner must buy PPE. The challenge is if the mine owner provides PPE, the new miner can disappear in 2 hours, and you buy again for the next employee” –* Mine owner, FGD

Biological hazards	*“There is something … faced in mines, insects such as mosquitoes get in the mines and bite people. Then rodents and rats come with ticks. There are other places named Ticks where ticks are in an area with gold, and people get attacked and injured by ticks” –* Mine owner, FGD *“Another point I had forgotten, people get bitten by snakes, snakes hide in timbering. Yes, yes, yes, we have had serious cases where people get bitten by snakes and die. There is also the problem of scorpion bites” –* Mine owner, FGD

Psycho-social hazards	*“Taking drugs like marijuana is common in ASM.” –* FGD with miners’ wives *“you get sick because of stress” –* Miner, FGD *“There is also the challenge with ‘Member’ (machete gangs) who raid and attack miners”* – Mine owner, FGD

**Themes**	**Examples of effects of safety and health issues: losses and fatalities**

Injuries, loss of ores, loss of body parts, and loss of ability to work	*“There is also the challenge with …. many people raid and attack miners and get other people’s ores. They can siege mine owners to injure and raid them. There are many cases like that” –* Mine owner, FGD *“Loss of ability to work” –* Male miner, 33 years old, IDI *“Common accidents at the mill involve loss of fingers when collecting the sands (milled ore) from the box*” – Male miner, 39 years old, IDI “*Injuries from a mine collapse in the rain season” –* Male miner, 33 years old, IDI One mill operator had an injured finger. He explained that he was fixing the hammer mill without gloves and was cut by loose parts of the hammer mill when he got injured. He acknowledged that the mine owner had provided gloves, but he was not using the gloves – Site observations

Fatalities	*“There was a guy who got into the shaft alone, was closed, and died” –* Male miner, 70 years old, IDI *“Shaking during blasting causes cracks, falling rocks, and collapsing mines, which can cause fatal injuries beyond rescue. Catastrophic injuries are common once in a while” –* Male miner, 29 years old, IDI *“Another point I had forgotten…, people get bitten by snakes, snakes hide in timbering. Yes, yes, yes, we have had serious cases where people get bitten by snakes and die. There is also the problem of scorpion bites” –* Mine owner, FGD *“…many people die of mine accidents and collapsing mines” –* Male miner, 70 years old, IDI *“Accidents and loss of life due to lack of skilled blasters” –* Male miner, 32 years old, IDI *“For now, we mine for 5–6 years, and we die. This causes artisanal miners to die. Artisanal miners are dying*’ – Miner, FGD

Accessibility of health services	*“We do not go anywhere at all. We go only when we are in severe pain” –* FGD with miners *”The hospital requires money; you have to raise your own money to go to the hospital, including money for transport. When you do not have [money] you just take paracetamol and keep working” –* Miner, FGD *“People can die without seeking health care. Delayed health-seeking is caused by transport challenges to get to the referral hospital” –* Miner, FGD *“It [accessing health care services] depends on whether you are injured or not. If injured, a police report is required.” –* Miner, FGD *”…. if one gets approval to go to Kadoma General Hospital, there is no medicine at the hospital. We used to get medicine at the dispensary within the hospital, but now there is no medicine at Kadoma General Hospital. One has to go to town to get medicine from the pharmacy.” –* Miner, FGD


## Environmental Hazards

The Gadza River in Shurugwi was dry in the rainy season. River siltation could be associated with water scarcity for agriculture and consumption. Some farmers in Shurugwi were also transitioning to ASGM on farming land due to persistent droughts. In Kadoma, some farmers discovered gold within their homesteads and started mining which was associated with chemical (mercury and cyanide) contamination of drinking water and farmland. However, mercury pollution is not further pursued in this study (***Tables 4*** and ***5***). There were no signs of reforestation, rehabilitation, or climate change awareness.

## Reported Experiences of Miners: Focus Group Discussions (FGDs) and In-Depth Interviews (IDIs)

As reflected below, numerous hazards in ASGM were confirmed in FGDs and IDI.

Qualitative data confirmed experiences of physical, chemical, biological, and psycho-social hazards. As shown in ***Table 5***, rock falls and mine collapses were reported as typical, especially in the rainy season. Sources of noise included blasting and drilling with jackhammers. Sanitation and hygiene were poor. There was no compliance to COVID-19 protocols on the majority of the visited sites. Reported effects included deaths, injuries, loss of ores, and loss of ability to work. The majority of the miners had no health coverage. Health seeking was delayed and denied due to lack of money and requirements to provide a police report in the event of accidents.

## Discussion

Findings revealed numerous hazards that required immediate attention; engineering controls and risk mitigation measures were missing, while PPE use was highly susceptible; to weaknesses and there was no prioritization of the hierarchy of controls. Almost 50% of survey participants had experienced health and safety challenges. Working underground, long working hours, and workplace violence were associated with experiencing health and safety issues at work. PPE was the common control measure; PPE was characterized by numerous weaknesses, which could result in increased adverse health, safety, and environmental issues. This section discusses the interaction between hazards and controls and the associated health and safety effects, with specific reference to PPE use, as well as missing engineering and risk mitigation controls.

Exposure to hazards with lacking and compromised PPE use demonstrated the vulnerability of PPE. While mine owners were required to provide PPE [5], the mine owners preferred individual miners (workers) to source their own PPE. Giving PPE to the miners was also challenged because of the migratory nature of ASGM, negative perceptions, and non-compliance, which is revealed in previous findings [25]. Simultaneously, the miners could not afford to buy PPE, ostensibly because of low earnings; this is supported by the literature [10,14,17]. Consequently, the replacement of PPE posed challenges in the sector. Smoking, alcohol abuse, and drug abuse were prevalent at work. This is interlinked with low compliance to PPE use [26]. It could be argued that PPE use was highly endangered with weaknesses of different shapes and sizes [12] (***Figure 1***) in the presence of numerous hazards. Compromised PPE use in ASGM could be associated with opening holes and weakening control measures [12], resulting in increasing odds of adverse health and safety effects [14].

Lack of effective control measures such as engineering controls undermines the safety and health of miners despite the prioritization of PPE [10]. Miners reported experiences of health and safety problems, including injuries, respiratory problems, and hearing problems, which have also been found in previous studies [14,15,16,17,18]. Wet crushing, noise-proof mechanisms, and securing standard shafts were lacking. Simultaneously, engineering control measures are associated with high costs [10], while ASGM is associated with low capital [1]. Hence engineering controls such as sinking standard safe shafts could be unaffordable for the majority of ASG miners in Zimbabwe because of low levels of capital. Concurrently, the odds of experiencing safety and health issues were two times higher when exposed to underground mining. In Zimbabwe, there has been a discussion among experts and stakeholders for regulations that address the needs and issues that are common in ASGM [27]. On the other hand, while the Environment Management Agency and Ministry of Mines and Mining Development have the mandate to offer training services to ASGM in Zimbabwe, ASGM has been growing fast, and there is limited capacity to train, implement, inspect, and monitor ASGM activities [28], thus increasing and opening more “holes” and “eyes” of various shapes and sizes in different positions in control measures [12]. Since ASGM is associated with limited capital, there is a need to address holes and weaknesses in the hierarchy of control measures by raising awareness and financing the prioritization of effective control measures.

Missing control measures threaten ASGM. In this work, defenses against hazards associated with sanitation and hygiene were compromised, as evidenced by lack of toilets, consumption of chemically contaminated water, and absence of hand washing facilities during COVID-19. Previous research has found sanitation and hygiene deficient in ASGM [15,16,17], which is associated with the spread of diarrheal diseases, tuberculosis, and malaria [14,16,17,18,29]. In addition, this study was conducted during the COVID-19 crisis, and the majority of the visited sites were not complying with defined COVID-19 protocols [30], pointing to missing control measures, in general. Health-seeking behavior was also low, partly due to lack of health coverage and the requirement to produce of an accident police report in order to access health services in the event of an accident. Access to health care services was limited [16,17] thus pushing some miners to resort to prayers/prophets, illegal drug sellers, and self-medication. Poverty and inaccessibility of health care centers are associated with low uptake of health care services [16,17].

Occurrences of fatalities in the absence of risk mitigation strategies exposed the miners. Mine collapses were common, especially in the rainy season. According to the McFarlane 2019 data set on ASGM fatalities, Zimbabwe experienced 42 ASGM fatalities in 2019 [31]. Working underground was associated with the double odds of experiencing safety and health issues. ASGM fatalities were therefore associated with negative outcomes for individual miners, their families, and ASGM communities in the absence of risk mitigation strategies. This is demonstrated by the case of a woman who lost her 34-year-old husband on December 8, 2020; the late husband was trapped underground while mining in the rainy season [32]. The widow had no compensation, no security cover or risk mitigation strategy. The majority of questionnaires respondents were married men, aged between 18 and 36 years, who were working underground. Underground fatalities entailed the loss of a young male workforce, which could contribute to adverse social, emotional, and moral impacts on families and ASGM communities [33]. The combined vulnerabilities due to exposure to numerous hazards without mitigation strategies could result in increased odds of experiencing health and safety issues and the intensification of adverse effects [34]. Hence, the relevance of risk mitigation strategies that address holes and weaknesses in control measures is clear, because they promote the health and safety of miners and ASGM families and communities [8,33].

Environmental threats were found in this study, which complements existing literature [1,13,14,15,31]. Washing of alluvial ores in rivers could contribute to the siltation of the Gadza River, which could result in water scarcity [13,14,15]. Underground mining, which is common in Zimbabwe, is also associated with heavy water consumption and falling water-table [35]. Water scarcity due to ASGM activities could contribute to community conflicts [35,39,40]. There is evidence of conflicts between farmers and ASG miners in Zimbabwe [28]. While persistent droughts had pushed some communities in Shurugwi into ASGM, increasing impacts of climate change such as heat stress and water scarcity are potential threats to [35] ASGM. Destruction of the ecosystem is also associated with increased respiratory diseases [36]. Administrative control measures such as awareness-raising on reforestation, rehabilitation, and climate change or planetary health were lacking.

Some scholars have viewed ASGM as an inherently dangerous and unsustainable sector [37] because of hazardous working conditions with compromised control measures. On the other hand, artisanal and small-scale mining has significantly contributed to the world’s mineral production, job creation [8,31] and livelihood sustenance [4]. In Zimbabwe, where ASGM has a significant economic role, the government and the international development community have supported the sector [6]. In 2012, the United Nations Environmental Program (UNEP) set the global initiative to reduce and, where feasible, eliminate mercury use in gold processing in ASGM [38]. Simultaneously, the international market and consumer certification approaches have set specific standards, including legitimacy, elimination of child labor, universal human rights, non-illegal involvement of security forces, and responsible mercury use as the benchmark for accessing fair international markets for gold from ASGM [39,40]. In Zimbabwe, there have been awareness-raising and routine inspections on PPE use in ASGM. However, as discussed above, there is no focus on comprehensive health and safety measures involving hazard identification and implementing adequate control measures. Hence the need to prioritize the hierarchy of control measures, which requires formalization of the sector in order to address health and safety issues in ASGM.

## Limitations

Data from the questionnaires, in-depth interviews, and focus group discussions were self-reported; recall and response biases were possible in reporting. However, direct hazard identification was also conducted while triangulation and the mixed method complemented strengths of qualitative and quantitative methods.

Findings may also have limited generalizability beyond Kadoma, Shurugwi, and Zimbabwe. Simultaneously, the study districts typify ASGM activities across Zimbabwe. This corroborates relevant studies on health and safety in ASGM. Therefore, the study may contribute significantly to ongoing research in the health and safety discipline in ASGM, both nationally and globally.

## Conclusion

Findings from this work confirmed numerous hazards inherent in ASGM in Zimbabwe and the need for these hazards to be addressed immediately. PPE was the common control measure, which was characterized by multiple weaknesses, while engineering controls and risk mitigation measures were missing. The interaction between numerous hazards, inadequate protection, and missing control measures was associated with adverse health, safety, and environmental effects. Numerous holes in PPE use and missing engineering controls and risk mitigation measures could be associated with at least a double increase of health, safety, and environmental effects when exposed to working underground, workplace violence, and long working hours. Therefore, it is imperative to prioritize the hierarchy of control measures in order to promote the health and safety of the workforce in ASGM.

## Recommendations

Financing and prioritizing observation of the hierarchy of control measures is indispensable to promoting the health of workers in ASGM. These control measures include securing safe shafts; wet blasting, wet crushing, and wet milling; managing noise levels; improving sanitation and hygiene; raising awareness on climate change; and increasing accessibility to health care.

## Additional Files

The additional files for this article can be found as follows:

10.5334/aogh.3621.s1 Appendices. Appendix 1–5.

10.5334/aogh.3621.s2 Data set. 20202 Health and Safety Survey among Artisanal and Small-Scale Gold miners in Kadoma and Shurugwi, Zimbabwe.

## Footnotes

Data Set [3].

Data Set [3].
