# Supplementary material for: Hazards and Control Measures among Artisanal and Small-Scale Gold Miners in Zimbabwe
Source: Ann Glob Health. 2022 Mar 15;88(1):21. doi: 10.5334/aogh.3621 (PMC8932352; doi:10.5334/aogh.3621)
Supplement: Appendices. — Appendix 1–5. [file agh-88-1-3621-s1.pdf]

Appendices

Appendix 1 Questionnaires

Assessment of the safety and health of workers and the relevant Occupational Safety & Health Management System in the Informal Sector: A Case of Artisanal and Small Scale Gold Mining in Zimbabwe and Uganda.

Site ....**Category**: ....**Date**…………………………………………………………………………

**Section 1: General Worker Information**	
**1.**Gender	□$_1$ Female □$_2$ Male
**2.** Age	□$_1$ 18–35 □$_2$ 36–50 □$_3$ >50 □$_{777}$Other Specify __________<br>□$_{999}$ Refuse to answer
**3.**Highest level of education	□$_1$ No formal school □$_2$ Primary □$_3$ Secondary □$_4$ Tertiary<br>□$_5$ Vocational □$_{777}$ Other Specify _______________<br>□$_{999}$ Refuse to answer
**4.** Knowledge and competence of your work in artisanal mining	□$_1$ Low □$_2$ Average □$_3$ High □$_{777}$ Other Specify________<br>□$_{888}$ Don't Know □$_{999}$ Refuse to answer
**5.**Marital status	□$_1$ Single □$_2$ Married □$_3$ Separated □$_4$ Divorced □$_5$ Widowed<br>□$_{999}$ Refuse to answer
**6.** Household size (Number of people staying within your family)	□$_1$ 1 □$_2$ 2-5 □$_3$ More than **5** □$_{777}$ Other, Specify _______________<br>□$_{999}$ Refuse to answer
**7.** Which assets have you owned through ASGM	□$_1$ Television □$_2$ Car □$_3$ Radio □$_4$ Bicycle □$_5$ Smart phone<br>□$_6$ House □$_{777}$ Other, Specify ____ □$_{999}$ Refuse to answer
**8.**Monthly household income	□$_1$ 100 USD and less □$_2$ 100-500 USD □$_3$ 500USD-1000 USD<br>□$_{777}$ Other, Specify _______ □$_{789}$ N/A □$_{888}$ Don't Know □$_{999}$ Refuse to answer
**9.**Have you moved and worked in different mining sites in the past 6 months	□$_0$ No □$_1$ Yes □$_{789}$ N/A □$_{999}$ Refuse to answer<br>If yes, please indicate why<br>……………………………………………………………………

**10.** Which department do you work in?	□$_1$Digging □$_2$ Blaster Blasting Licence□$_0$ No □$_1$ Yes □777 Other, Specify _______<br>□$_3$ Lashing □$_4$ Washing □$_5$ Carrying/moving mined ore to the surface<br>□$_6$ Loading/off when transporting ore to the stamp mill □$_7$ Sponsor<br>□$_8$Manager/Supervisor/Gang leader □$_9$ Working at the Stamp Mill □$_{10}$Mine Owner<br>□$_{11}$ Amalgam burning □$_{12}$Cooking □$_{13}$ Carrying ore manually to the stamp mill<br>□ $_{14}$ Gold buying<br>□$_{777}$Other, specify _____ □$_{888}$ Don't Know<br>□$_{999}$ Refuse to answer □$_{789}$ N/A<br>**a)** Do you work underground?<br>□$_0$ No □$_1$ Yes □$_{789}$ N/A □$_{888}$ Don't Know □$_{999}$ Refuse to answer<br>**b)** If yes do you work alone?<br>□0 No □$_1$ Yes□789 N/A □$_{888}$ Don't Know □$_{999}$ Refuse to answer<br>**c)** How many hours do you work in a day?<br>□$_1$ 1-8 □ $_2$ 9-16 □$_3$ 17-24 □$_{888}$ Don't Know □$_{999}$ Refuse to answer<br>**d)** What kind of working arrangement do you have<br>□$_1$ Salary □ $_2$ Shares □$_3$ Contractor □$_{777}$Other, specify _____<br>□$_{888}$ Don't Know □$_{999}$ Refuse to answer
**11.** How long have you worked in artisanal and small scale gold mining?	□$_1$1–5 years □$_2$6–10 years □$_3$11–15 years<br>□$_4$ > 15 □$_{789}$ N/A □$_{666}$ Don't Remember □$_{999}$ Refuse to answer
**12a)** Do you know of HIV/AIDS test center close to your work place	□$_0$ No □$_1$Yes □$_{789}$ N/A □$_{888}$ Don't Know □$_{999}$ Refuse to answer<br>**b)** Have you ever taken HIV test?<br>□$_0$ No □$_1$ Yes □$_{789}$ N/A □$_{888}$ Don't Know □$_{999}$ Refused to answer<br>**c)** If yes and you are comfortable, may you share your status?<br>□$_0$ Negative □$_1$ Positive □$_{789}$ N/A □$_{888}$ Don't Know<br>□$_{1010}$Uncomfortable to answer
**13a)** Have you ever experienced any health problems, became sick or got injured because of mining activities?	□$_0$ No □$_1$ Yes<br>b) If yes, please specify,<br>□$_1$ Respiratory problem□$_2$ Memory problems □$_3$ Skin problems<br>□$_4$ Reproductive problems □$_5$ Hearing problems □$_6$ Musculoskeletal Problems<br>□$_7$ Kidney problem □$_8$ Sight problems□$_9$ Digestive problems □$_{10}$Stress

	□11 Injury □777 Other Specify ______□789 N/A □888 Don't Know<br>□999 Refuse to answer
**14.** How would you rate your health today from 1 to 100? **1 is the worst 100 is the best health state______**	
**Section 2 Occupational Safety**	
**15**. Who is responsible for safety on your site	□1 Mine Owner (on site) □2Supervisor □3 Gang leader □4 Employee □777Other Specify__________ □789 N/A<br>□888 Don't Know □999 Refuse to answer
**16a)** Have you ever had safety issues at your workplace	□0 No □1 Yes<br>b) If yes, in which department/area?<br>□1Going down to the mine/returning to the ground<br>□2Excavation □3 Blasting □4Lashing □5Crushing<br>□6Removal of ore from shafts □7Milling □8Sluicing<br>□9Loading, carrying of ore to the mill and off loading<br>□10Amalgam burning □11Delivery and selling of gold<br>□12Acquisition and delivery of equipment and chemicals<br>□13Storage of equipment and chemicals □14Disposal of waste<br>□777Other, specify _________ □789 N/A □666Don't Remember<br>□999 Refuse to answer<br>**c) What was the safety issue?**<br>□1 Slipping /tripping/ falling □2 Hit by working tools/machines □3 Instant death<br>□4 Hit by pieces of stone □5 Breaking rope (hoist) □6 Collapsing<br>□7Mine shaft collapse<br>□8 Failing to breath □9 Trapped underground □999 Refuse to answer<br>□777Other, Specify________________ □789 N/A □888 Don't Know
**17.** When an accident occurs at work how is it handled?	□1 Reported to area chief □2 Reported to County Director of Mines<br>□3 Reported to Country Commissioner □4Not reported<br>□5Reported to the hospital □777 Other Specify________________<br>□789 N/A □888 Don't Know □999 Refuse to answer
**18.** What actions are	□1 Investigation into the cause □2Temporary Mine closure □3None<br>□777 Other, specify ________________□789 N/A □888 Don't Know

normally taken after an accident?	□$_{999}$ Refuse to answer
**19 a).**<br>Have you ever been injured in the course of your work?	□$_{0}$ No □$_{1}$ Yes<br>b) If yes, what was the type of injury?<br>□$_{1}$Cuts □$_{2}$ Fractures □$_{3}$Bruises □$_{4}$ Back/Chest injuries<br>□$_{777}$Other, specify__________ □$_{789}$ N/A □$_{666}$Don't Remember<br>□$_{999}$ Refuse to answer
**20a).** What was the cause of the injury?	□$_{1}$ Struck / hit by rock □$_{2}$ Sharp (rock) edges □$_{3}$ Fall □$_{4}$Vibration<br>□$_{5}$Working tools/Machinery □$_{6}$Lifting heavy load □$_{7}$Awkward posture<br>□$_{777}$ Other, specify ________ □$_{789}$ N/A □$_{888}$ Don't Know □$_{999}$ Refuse to answer<br>**b)** Which part of your body was injured<br>□$_{1}$ Head □$_{2}$ Legs □$_{3}$ knee □$_{4}$Feet □$_{5}$ Back □$_{6}$ Chest □$_{7}$ Hand □$_{8}$ Palm<br>□$_{777}$ Other, specify_______________ N/A □$_{888}$ Don't Know □$_{999}$ Refuse to answer<br>**c)** Do you have access to compensation<br>□$_{0}$ No □$_{1}$ Yes□$_{789}$ N/A □$_{888}$ Don't Know □$_{999}$ Refuse to answer<br>□$_{777}$ Other, Specify_______________<br>**d)** Do you have access to health care insurance<br>□$_{0}$ No □$_{1}$ Yes □$_{789}$ N/A □$_{888}$ Don't Know □$_{999}$ Refuse to answer<br>□$_{777}$ Other, Specify_______________
**21a).**<br>Have you ever heard about	No □$_{0}$ Yes□<br>**b)** If yes, where did you get the information?<br>□$_{1}$ Fellow miner □$_{2}$ Mine Site Manager □$_{3}$ Radio / TV<br>□$_{4}$ Friend / Family □$_{5}$ Social media (Facebook / Twitter)

workplace safety and health?	□6 Newspaper / Magazine □7Training □ 789 N/A<br>□777 Other, specify________□666 Don't Remember □999 Refuse to answer
**22. a)** Do you use personal protective equipment (PPE) when working?	No □0 Yes□1<br>**b)** If yes, which PPE?<br>□1 Helmets □2 Protective footwear □3 Protective glasses /face masks<br>□4 Protective clothing(jackets) □5Ear protection □6 Dust mask<br>□7Respirators □8Protective belt □9Sunhat □10Raincoats<br>□777 Other, specify _______________ □789 N/A<br>□999 Refuse to answer<br>**c)** How did you get the PPE?<br>□1 Provided by site manager □2 Bought for myself □3 Given by co-worker<br>□4 Given by a friend / family □777 Other, Specify___________ □789 N/A<br>□999 Refuse to answer<br>**d)** How often do you replace your PPE (dust masks, ear plugs, etc.)?<br>□1 FREQUENTLY □2 Rarely □3 Never □789 N/A □999 Refuse to answer<br>**e)** What are the common reasons for not using Personal Protective Equipment?<br>□1 Not provided □2 Not required □3 Not comfortable □4 Not affordable<br>□5 Not aware of their importance □789 N/A □888 Don't Know<br>□777 Other, specify_______________ □999 Refuse to answer
**23**.Have you ever experienced a case(s) of violence related to your work?	□0No Yes □1 □888 Don't Remember<br>If yes, what kind of violence<br>□1People taking over shafts by force □2 Raiding of ore, gold, equipment etc.<br>□3 Physical attacks □4 Fights at work □5 Gender based violence<br>□777 Other [Specify]<br>____
**23a)** Have you ever been trained on OSH and/or first aid?	**OSH** □0No Yes □1 First **Aid** □0No Yes □1<br>**b)** If yes, who was the trainer?<br>□1 Mine /Site Manager(supervisor)<br>□2 Safety expert □3 Workmate □4 NGO □777 Other, Specify_______________<br>□789 N/A □999 Refuse to answer
**24.** Which	□1Occupational Safety and Health training □2Financial support

services do<br>you have<br>access to	□$_3$Mining services □$_4$ Shops with PPE □$_5$ Health Care<br>□$_6$ Social security □$_7$ Emergency Services □$_8$Competitive gold markets<br>□$_9$ Schools □$_{10}$ Partnerships/Associations<br>□$_{11}$ Communication Specify □TELEPHONE □RADIO □TELEVISION<br>□9 NEWSPAPER □SOCIAL MEDIA □ COMMUNITY GATHERINGS<br>□$_{777}$ Other services, Specify_______ □$_{789}$ N/A □$_{888}$ Don't Know<br>□$_{999}$ Refuse to answer
**25.** What do you consider the priority safety and health issues at your work place?	□$_1$ Dust □$_2$ Noise □$_3$ Flooding □$_4$ Injuries/accidents □$_5$Hot confined shaft<br>□$_6$ Workplace Violence □$_7$Muscle and back pain □$_8$ Explosive fumes □$_9$ TB<br>□$_{10}$HIV□$_{11}$Mercury burning □$_{12}$Malaria □$_{13}$Snakes □$_{14}$ Hunger<br>□$_{15}$ Diarrheal diseases □$_{16}$Heat Stress □$_{17}$Cyanide □$_{777}$Other, Specify _____<br>□$_{789}$ N/A□$_{888}$ Don't Know □$_{999}$ Refuse to answer
**Section 3 Standard Operating Procedures**	
**26**. Do you have specific ways of working safe before during or after when performing activities like entering the mining pit, drilling, working with explosives lashing after blasting with explosives, manual lifting or carrying heavy loads, burning amalgam, milling or tanks/ cyanidation	□$_0$No □$_1$ Yes $_{888}$ Don't Know<br>If yes, please list them here____<br>____<br>____<br>____<br>____<br>____<br>____<br>____
**Section 4. Gendered Challenges**	
**27**. Would you indicate roles for men and women in the following activities<br>Male<br>Female<br>Both	Prospecting (prospector/sampler) □ Male □ Female □ Both<br>Digging □ Male □ Female □Both<br>Drilling □ Male □ Female □Both<br>Blasting □ Male □ Female

	☐Both<br>Lashing ☐ Male ☐ Female ☐ Both<br>Transportation of ore ☐Male ☐ Female ☐ Both<br>Crushing ☐ Male ☐Female ☐ Both<br>Milling ☐ Male ☐ Female ☐ Both<br>Sluicing ☐ Male ☐ Female ☐ Both<br>Washing/Panning ☐Male ☐ Female ☐ Both<br>Amalgamation ☐ Male ☐ Female ☐ Both<br>Cyanidation ☐ Male ☐ Female ☐ Both
**28.** Are there workplace challenges/difficulties common for men or women at your work?	Men ☐$_0$No ☐$_1$Yes Women ☐$_0$No ☐$_1$ Yes<br>If yes, please specify:<br>Men____<br>____<br>____<br>_______________<br>If yes, please specify: Women<br>____<br>____<br>_______<br>____<br>_______

Appendix 2 Hazard identification and risk assessment tool [24.25]

Hazard Identification & Risk Assessment

Item	Hazard identified	Associated risk related to hazard	Probability index	Severity index				Frequency index	Risk score	Risk value
				**I**	**P**	**E**	**C**			

Key
I: Injury
P: Impact of risk on production
E: Impact of risk on the work environment
C: Associated Cost of the risk

Appendix 3 Weighted Risk matrix of 1-5 based on probability; the severity on injury/diseases, the severity on production, the severity on the environment, the severity on cost; and frequency; modified after Basgen and Baudeee et al.[23,24]

**Probability Index**	**Severity** **Injury/Disease (I)**	**Severity** **Production P**	**Severity** **Environment E**	**Severity Cost (C)**	**Frequency**
**(5)** Very high	**(5)** Fatal	**(5)** No production (Loss of > month)	**(5)** Permanent effects	Very expensive	**(5)** Hazard permanent Present
**(4)** High	**(4)** Permanent to slight disability	**(4)** Loss of > 1 week to 1 month	**(4)** Long term > 2 years	Expensive	**(4)** Hazard arises weekly
**(3)** Moderate	**(3)**> 14 Days recovery	**(3)** Loss of 1 week	**(3)** Medium > 6 months<br>to 2 years	Average	**(3)** Hazard arises monthly
**(2)** Minor	**(2)** Medical attention<br>full recovery<14 days	**(2)** Loss of 1 day	**(2)** Short term 1 day to 6<br>Months	Cheap	**(2)** Hazard arises yearly
**(1) Unlikely**	**(1)** First aid only	**(1)** Loss of 1 shift	**(1)** Insignificant effect	Very cheap	**(1)** Hazard arises in 5 years

Appendix 4 Focus Group discussion questions

Focus Group Discussion: Men and women ASGM miners

1. a) Do you have knowledge of relevant Acts and Regulations on OSH?
   b) What do you know about health and safety at work?

2. a) Could you share your experiences with accidents at work?
   b) From your experience how many days have you lost at work because of accidents?
   c) How are you and your families affected by these accidents?
   d) How is your production and earnings affected by such accidents?

3. In the above context

i. Would you say it is necessary to protect your health and safety**?**
ii. Which safety and health practices do you have?
iii. How do you burn amalgam (retort, open in the air, at home under closed doors?
iv. How do you store mercury and the equipment and PPE/clothes you use when burning amalgam?
v. Which measures do you usually follow during blasting?
vi. Which measures do you usually follow during cyanidation?
vii. Which measures do you usually follow when using equipment?
viii. Which measures do you usually follow when developing mining pits and mining?
ix. Where do you get water to drink during working hours?
x. Are you able to use toilets during working hours?
xi. What is the culture on changing the hoist rope?
xii. Which aspects of safety practices do you think needs improvements at your work? Please explain.

2. What is the health and safety issues affecting women in ASGM?

3. How is the health of your family and children affected by your involvement in ASGM?

a) Have you ever had a disabled child/ child with slow development since you started working in artisanal small scale gold mining?

b) What are the issues affecting women and children at your workplace?

c) Is your sexual life affected by your work?

4. **a)** Do you think it is necessary/ useful to have safety and health management a work?

b) If so, please explain.

c) If no, please explain.

d) Who should be responsible for the management of health and safety in the informal sector?

5. Do you think it is possible to extract gold without mercury (mercury free technology) at your work to protect the health of the workers?

6. What are the common perceptions on mercury use and the management of safety and health (mercury use/ mercury free technology, shaft requirements)?

Explosives and blasting requirements, PPE etc.) at your work

7. What do you think are the existing gaps and challenges in managing workplace safety and health at your site (reducing mercury use/ mercury free technology, requirements on shafts, explosives, blasting, fire extinguishers, first aid, PPE)?

8. a) What do you think are the factors/issues to address to improve management of OSH in the informal sector e.g., ASGM?

b) What do you think are the factors/issues to address to improve the compliance to relevant regulations on OSH in the informal sector (ASGM)?

**9.** What are your suggestions on managing safety and health in artisanal and small scale gold mining (mercury use/ mercury free technology, shaft requirements, explosives and blasting requirements, PPE etc.? Would you say the same safety and health regulations in large mines could be applied to artisanal and small scale mines to improve the safety and health of workers in ASGM?
10 a) Would you explain how your work was affected by the coronavirus pandemic?<br>b) What challenges did you face on the management of your health and safety at work during the corona virus pandemic?<br>c) What is needed for your workplace to be able to recover from the impacts of Corona Virus?<br>d) What do you suggest to be done for OSH management in terms of disaster preparedness and response in ASGM for emerging infectious diseases such as the Corona virus?
**11**. Where do you see Artisanal Gold Mining in communities in relation to climate change challenges such as drought?

2. a) Do you have knowledge of relevant Acts and Regulations on OSH?<br>b) What do you know about health and safety at work?<br>2. a) Could you share your experiences with accidents at work?<br>b) From your experience how many days have you lost at work because of accidents?<br>c) How are you and your families affected by these accidents?<br>d) How is your production and earnings affected by such accidents?<br>3. In the above context<br>xiii. Would you say it is necessary to protect your health and safety**?**<br>xiv. Which safety and health practices do you have?<br>xv. How do you burn amalgam (retort, open in the air, at home under closed doors?<br>xvi. How do you store mercury and the equipment and PPE/clothes you use when burning amalgam?<br>xvii. Which measures do you usually follow during blasting?<br>xviii. Which measures do you usually follow during cyanidation?<br>xix. Which measures do you usually follow when using equipment?<br>xx. Which measures do you usually follow when developing mining pits and mining?<br>xxi. Where do you get water to drink during working hours?<br>xxii. Are you able to use toilets during working hours?<br>xxiii. What is the culture on changing the hoist rope?<br>xxiv. Which aspects of safety practices do you think needs improvements at your work? Please Explain
**2.** What is the health and safety issues affecting women in ASGM?
**3.** How is the health of your family and children affected by your involvement in ASGM?<br>**d)** Have you ever had a disabled child/ child with slow developme<br>**e)** nt since you started working in artisanal small scale gold mining?<br>**f)** What are the issues affecting women and children at your workplace?<br>**g)** Is your sexual life affected by your work?
**4. a)** Do you think it is necessary/ useful to have safety and health management a work?<br>**b)** If so, please explain<br>**e)** If no, please explain<br>**f)** Who should be responsible for the management of health and safety in the informal sector?

**5.** Do you think it is possible to extract gold without mercury (mercury free technology) at your work to protect the health of the workers?
**6.** What are the common perceptions on mercury use and the management of safety and health (mercury use/ mercury free technology, shaft requirements)?<br>Explosives and blasting requirements, PPE etc.) at your work
**7.** What do you think are the existing gaps and challenges in managing workplace safety and health at your site? (reducing mercury use/ mercury free technology, requirements on shafts, explosives, blasting, fire extinguishers, first aid, PPE
**8. a)** What do you think are the factors/issues to address to improve management of OSH in the informal sector e.g. ASGM<br>**b)** What do you think are the factors/issues to address to improve the compliance to relevant regulations on OSH in the informal sector (ASGM)?
**9.** What are your suggestions on managing safety and health in artisanal and small scale gold mining (mercury use/ mercury free technology, shaft requirements, explosives and blasting requirements, PPE etc?<br>Would you say the same safety and health regulations in large mines could be applied to artisanal and small scale mines to improve the safety and health of workers in ASGM
10 a) Would you explain how your work was affected by the Corona virus pandemic<br>e) What challenges did you face on the management of your health and safety at work during the corona virus pandemic?<br>f) What is needed for your workplace to be able to recover from the impacts of Corona Virus?<br>g) What do you suggest to be done for OSH management in terms of disaster preparedness and response in ASGM for emerging infectious diseases such as the Corona virus?
**11**. Where do you see Artisanal Gold Mining in communities in relation to climate change challenges such as drought?

Appendix 5: In-depth interview questions

Describe your usual working day	What are your health needs at work	Describe what could be dangerous to your health at your workplace	Which PPE do you have at your workplace	Would you use PPE if you had a choice	What are the challenges with using PPE	What is the impact of mining on your health and your daily life	Possible health interventions
