# Supplementary material for: Hazards and Control Measures among Artisanal and Small-Scale Gold Miners in Zimbabwe
Source: Ann Glob Health. 2022 Mar 15;88(1):21. doi: 10.5334/aogh.3621 (PMC8932352; doi:10.5334/aogh.3621)
Supplement: Data set. — 20202 Health and Safety Survey among Artisanal and Small-Scale Gold miners in Kadoma and Shurugwi, Zimbabwe. [file agh-88-1-3621-s2.zip › s2-agh-3621_singo/Data _Set_3_FGD_Transcripts_Dat.pdf]

Focus group 1 Kadoma (2017) men and women miners

F: When looking at the way you work including other artisanal miners, how is the health situation in artisanal how is the health situation in relation to Occurrences of sicknesses in relation to how you work in artisanal mining?

1R: As we are working now there is no one who has faced sicknesses due to working conditions.

F: I understand, are there things like exposure to dust?

2R $_1$: There is dust exposure but we encourage all the workers exposed to dust to use respirators. So we haven't has experienced health challenges due to dust exposure.

F: Thank you, how about in other mines, are the miners being affected by exposure to dust.

3R: For me, I haven't come across a case of TB contracted by a miner due to dust exposure at work here.

F: What else are you doing to avoid accidents and injuries in artisanal mining?

4R: Where I work we encourage avoiding unsafe working environments to avoid injuries.

Dangerous instruments and tools not in use should be cleared and kept away to

Keep the working area safe and avoid tripping

F: How often do you do safety talks?

5R: Every day before starting work we have talks on the type PPE one should use. Checking the PPE for snakes and scorpions before wearing PPE. There could be a snake or scorpion hiding in the gumboots; one must therefore check what could be hiding in the gumboots before wearing.

F: You doing a good job. When it comes to accessing health care are there challenges in terms of getting transport to the hospital and accessing health care at the hospital.

6R: The Company normally helps. For example, if one gets sick, the worker can be given money by the company to go to the hospital and should provide evidence of hospital records and proof of medical payments made.

F: Good, how about others, is there any challenges or difficulties in getting medical care from hospitals?

7R: I don't think there is any problem.

F: How about mother?

8R: We do mining. Before mining, the leader should go down to check on safety as well as on the timbering. If not safe, safety measures are taken, after which the miners can go down and work.

F: When it comes to blasting what do you do to protect health?

9R: There is a blaster, it takes the blaster to explain.

F: How about when you need transport to get someone to the hospital?

10R: There are company cars and transport is normally there.

F: When you get to the hospitals do you get all supplies?

11R: It depends on the approved hospital depending on your health problem. You can get for a private approval hospital for serious problems or public hospitals for minor ailments such as general influenza.

F: That's good. What do you think is needed for artisanal miners to work in good health?

12R: We encourage use of gumboots, work suits and helmets, hard hats protect the head from injuries. Where we work there at the mill there is the risk of falling objects, with a helmet, if something falls on your head, you won't get injured.

F: How about you what do you do?

13R: We use work suits, safety shoes and respirators.

F: About PPE are challenges with working with PPE?

14R: Here at the mill there are no challenges.

F: Some miners are saying gumboots are too hot and can cause heat blisters while masks make it difficult to breath. What is your experience with using PPE?

15R$_1$: Here it's a must to always wear PPE, because if you are at work you are expected to wear PPE until the end of your 8 hours.

16R$_2$: We wear because it's safety.

F: Thank you very much. Have you ever heard of the Minamata Convention, a United Nations Program to find ways of minimizing mercury use in artisanal gold mining? What do you think about mining gold without mercury?

17R: I think it's challenging because mercury is what is use to extract the gold. I don't know it's if it's possible but I think mercury is needed to extract the gold.

F: What are your views, on the impacts of mining gold without using mercury?

18R$_1$: It's difficult because, mercury is what is used to extract gold.

19R$_2$: Mercury is the money; mercury is what makes gold extracted.

20R$_3$: If we don't use mercury, all the gold will be lost, all of it goes away.

21R$_4$: You get no gold without mercury.

F: What if you are shown alternative ways which are effective, like the mercury free technology.

22R: It depends on how the technology works. We are convinced after seeing how it works. At the moment we are convinced that if we use mercury we extract all the gold.

F: The United Nations expects to eliminate mercury use in artisanal mining. In the event that mercury use in artisanal mining is eliminated how could it affect artisanal mining?

23R$_1$: It depends on how the technology works on whether the alternative methods have the same efficiency as mercury.

24R$_2$: If people are convinced that will be acceptable.

F: What if it's passed as a law, how does it affect your work?

25R$_1$: We stop mining because if one mines fine gold, fine gold can't be extracted without mercury "*rinongosvika richirasika*" (the gold gets lost).

26R$_2$: The owners of milling plants will gain, we miners will not gain, and the owners of the ore won't get anything.

F: In the current situation where people are still using mercury, how can mercury be used while protecting people's health?

27R: People must use respirators when handling mercury.

F: But do people use respirators. It has been expressed that respirators make it difficult to breath. Is there anyone who has experienced how the respirators work?

28R: There are variations among respirators; some respirators are too tight while the other respirators are comfortable. It's just like gumboots; gumboots must leave some space, if gumboots are too tight they can cause blisters.

F: So you are saying there are some respirators which allow comfortable breathing?

29R: Yes.

F: Have you ever spent a day wearing a respirator?

30R_1: Yes.

31R_2: I used to do it when I was working at Rio-Tinto.

F: Have you ever had the pressure to remove the respirator?

32R: Removing the respirator could have been harmful to me. How could I take off the respirator in that dust and chemical polluted environment? One would rather persevere with the dust until the job is done.

F: What are your views on the use of the mutton cloth in place of the respirator?

33R: Mutton cloth is good, just like respirators, when one ties mutton cloth, dust won't get into the lungs.

F: How about using the mutton cloth against chemicals?

34R: You only need to make the mutton cloth wet, not knowing if there could be harm with mercury vapor, it might be possible that some mercury could still get into the body which can only be proved with testing mercury contamination.

F: How about you, you wanted to add something?

35R: Yes, mercury contamination is more dangerous at amalgam burning, from the white mercury vapor. There is a system called the retort pot where the amalgamation is enclosed and the mercury vapor is condensed back into mercury and no vapor is

inhaled. The challenge is during concentration because we cannot wear gloves during concentration, gloves can get torn and there will be contact with mercury.

F: In other places the retort was introduced and resisted because people were used to mercury. Do you think the retort could be acceptable here in Kadoma?

36R: Retort is good. One the mercury you use is recycled and you are protected from mercury vapor. You set the retort and do amalgamation away from people. The challenge is the retort takes more time and it's difficult to use it here at the mill because the miners want to get there gold faster.

F: But does everyone understand the retort?

37R: For the miners, it's difficult to make them understand. If the owners of the gold miners understand the process, it will be better and it takes time to make the miners understand.

F: In terms of sicknesses are there local names, for different sicknesses and local practices. Are there local names for tuberculosis, mercury poisoning, injuries,

38R: No.

F: How about TB, there is a talk on bone TB, how is it different from other types of TB.

39R: Might not be found in lungs but will be in bones.

F: How about management of sicknesses, are there local practices?

40R: We encourage people to go to the hospital.

F: How about others?

41R: It's personal; people know the life they are in. Some people say they don't go to hospital; they belong to apostolic sects which do not approve going to hospital so it depends on people.

F: So those who say they don't go to the hospital sort medical attention from churches?

42R$_1$: Yes, so when they get sick they say take me to my church. May be when it fails they can go to the hospital.

43R$_2$: Some people believe that if they get sick they can get well after church prayers, but it's different when someone's hand is broken, so we encourage people to go to the hospital when they get sick.

F: Some people talk of drinking '*maheu'* traditional commercial beer to clear the dust after dust exposure.

44R: After getting exposed to lots of dust, drinking maheu helps clear the residual dust.

F: Is there anything else people are doing to protect their health?

45R: Some people drink milk or mazoe (local drink beverage) after dust exposure but maheu is better its thick so when you swallow it rubs off and clears the dust on the throat.

F: Some people talk of drinking *'muti'* (traditional medicine) to be strong. Have you ever heard of that?

46R: It's traditional and it's everywhere, people normally take *'muti'* to keep their bodies strong.

F: Are there other common traditions. In other places people pray some tie strings on their bodies for protection. Are there any other practices observed here?

47R: We come from different backgrounds and different homes. People could observe some rituals before coming to work. But here at work we encourage people to go to hospital.

F: Thank you for your time. Is there anything else you would like to say?

48R: On the previous question on accessing health care, if one gets approval to go to Kadoma General Hospital, there is no medicine at the hospital. We used to get medicine at the dispensary within the hospital but now there is no medicine at Kadoma General Hospital. One has to go to town to get medicine from the pharmacy. We desire to have medicine at the hospital at the dispensary and blankets. There are no blankets at the hospital.

F: In other places miners expressed concern in the fact that if you tell the doctor that you are mining, the doctor gets emotional and advices to stop mining, is it the same here?

49R: We haven't met challenges; normally we are treated like an ordinary person

F: Well thank you very much, any other additions?

50R: No, there are no other additions.

F: Thank you for your time.

51R: We also appreciate.

Focus Group 2 Kadoma (2017) Men

F: We would like to discuss how artisanal miners could work while protecting themselves; I can see that some of you are wearing protective clothing.

52R$_1$: What is needed is to protect health is to wear safety clothes, gumboots, helmets, work suits, touches. Gloves protect the hands; we work with stones.

53R$_2$: When drilling there is need to cover the mouth and nose because dust from drilling can affect health like the lungs and TB.

F: You have raised important issues like TB. Are there other health issues? You come across in mining.

54R$_1$: People are more affected if there is no protective clothing.

55R$_2$: Injuries are common in shafts which are not strong not strong. There is need to check if there are falling rocks, pulling them down leaving the shaft safe.

F: Are there other issues?

56R: No.

F: In other places local names are given to health issues like bone TB, are there local names used for health issues here?

57R: No

F: How about bone TB. Have you ever heard of bone TB?

58R: No.

F: Alright, so when people get sick here do you they go to the hospital, or sicknesses are managed at home, what happens when people get sick?

59R$_1$: We buy tablets.

60R$_2$: We also get left over tablets from someone which were left by someone like paracetamol. Some people keep paracetamol because they cannot afford to go to private hospitals because private hospitals are more expensive.

F: So what is needed for people to access health care?

61R: Normally there is need for cash for consultation.

F: How about reaching to the hospital is there need for transport or health centers are close.

62R: There is need for transport to get to the health Centre.

F: Thank you very much. You may leave and go back to your work and I continue with the other group.

Group B

F: So which sicknesses do you come across as you mine?

63R: Coughing, dust especially from using the jack hammer.

F: So where do you go to seek health care, is it nowhere at all?

64R: We do not go anywhere at all. You go only when you have intense pain.

F: Do you have other ways to manage sicknesses or to get healing?

65R: When you are in intensive pain you go to the hospital.

F: How is the situation when you get to the hospital? Are you able to get medical attention when you get to the hospital?

66R: You must have the money.

F: How do you get the money?

67R: Here at work we don't get paid, when you get sick it's difficult to get to the hospital. You can sell your possessions to get money to pay for medical bills.

F: But when you get to the hospital do you get the medicine.

68R$_1$: Medicine will be there but money is needed to buy the medicine.

69R$_2$: The government hospital Centre normally doesn't have the medicine and health care professionals normally refer you to pharmacies to get medication.

F: How about you, how do get medical care?

70R$_1$: The hospital requires money; you have to raise your own money to go to the hospital, including money for transport. When you don't have you just take paracetamol and keep working.

71R$_2$: You also need money for transport to get to the hospital.

F: Is there anything else needed to access health care?

72R: No.

F: So if you look at the work how you could work while protecting your health?

73R: Respirators, gumboots, gloves, helmets: all safety clothing.

F: Which PPE, do you have at the moment?

74R: At the moment we don't have. We get into the shaft like this, without PPE. When we get money, we should buy for ourselves.

F: Is PPE affordable?

75R: It's not expensive. The only challenge is rent is also needed. The money you get also needs to pay for rent.

F: Some miners are saying even if they the get PPE, PPE is too difficult to use underground. If one wears complete PPE, it's difficult to work underground. What are your views and experiences with PPE?

76R$_1$: It depends on how one feels when wearing PPE.

77R$_2$: It's because there is water in the shaft, if you get wet PPE wraps over your body in uncomfortable way which makes it difficult to work.

F: What are the views of others?

78R$_1$: My view is PPE is still needed.

79R$_2$: What happens PPE makes you sweat, and some people have High blood pressure so wearing many clothes can make breathing difficult for them?

80R$_3$: Like gloves, not many people are used to working with gloves but things like gumboots, work suits, helmets one could wear.

F: For you what would you like to add?

81R: The issue is when hitting with the hammer, you hit hard and sweat and it's preferred for one to be free without PPE, because one will be working hard.

F: Some miners are saying wearing masks closes the mouth and makes breathing difficult. What is your view on working with respirators?

82R: Respirators are needed when drilling, drilling takes about 30 minutes. There is no need to wear masks for a long time. Respirators are needed only for the drillers

F: After drilling there are normally gases from drilling. Masks could be used during digging and lashing after drilling. Other miners were saying its burdensome to work with the respirators after drilling. How is it for you?

83R$_1$: For that respirators are needed because of the fumes after blasting.

84R$_2$: Closing the mouth and the nose makes breathing difficult, there is need to breath enough air.

R$_3$: You don't breathe well with respirators; you need to get enough air.

85R$_4$: Respirators are needed because those fumes from blasting can cause problems like TB. When the fumes are still there, there is need to wear respirators. Respirators are needed they must not running. We are not using respirators because of lack.

F: So for you can you say if you get the respirator you are going to use it?

86R: Yes.

F: So for you, are you saying respirator is important when burning amalgam.

87R$_1$: Yes.

88R$_2$: Respirators are needed; the fumes from blasting are harmful. Respirator is needed, our job is risking, even with blasting, you must get in after a specific time after blasting but with us our work 'chikorokoza' 'informal' you get in just after blasting when the fumes are still there, so the respirator is still needed. One can get a headache or

get difficulty in breathing when getting into the shaft soon after blasting; you just persevere for the work to move on.

F: So how many hours should you wait to get into the shaft after blasting?

89R: The law says 3-4 hours but here we wait just for 30 minutes and we get in

F: So you are saying you use the mutton cloth when you don't have the respirator?

90R: Yes, you won't be having the respirator.

F: Do you think the respirator is needed?

91R: Yes, the respirator is needed.

F: So if you get the respirator, are you going to use it?

92R: Yes

F: Wood you use the respirator here and there or every time

93R: Here and there

F: When would you use the respirator?

94R: When I can see and smell the blasting fumes, I can take the respirator off when the fumes have cleared.

F: Thank you very much. About the use of mercury in processing gold. How do you view the use of mercury? Do you say it affects your health or not?

F: How do you view use of mercury in gold mining? Do you think it can affect health or you say it cannot affect health?

95R_1: Mercury is the one that 'catches' gold

96R_2: Mercury doesn't affect our health in any way. It makes us health because it makes us get money

97R_3: Ah! Mercury is harmful, just like acid; mercury helps you get the gold but causing harm on your health.

98R$_4$: The time you burn amalgam, that mercury vapor can be harmful

99R$_5$: No, everything is harmful there is nothing not harmful

100R$_6$: But for us it's only because we are struggling it's the situations we are going through which make us force ourselves to do dangerous things because there is no option

F: How about you, what do you say?

101R: Mercury can affect health

F: Have you ever heard of what you could do to reduce use impacts of mercury

102R$_1$: We haven't heard of anything

103R$_2$: You must stand aside and not directly face the mercury vapor during amalgam burning

F: Have you ever heard of the retort?

104R$_1$: No

105R$_2$: The respirator is needed when using burning amalgam.

F: How about others, what do you think?

F: If it's in the open, I can use the respirator. The respirator protects a lot

F: So if you get the respirator do you use it

106R: Yes

F: So we are saying the respirator is also important to protect our health against mercury?

107R: Yes

F: But others are saying mutton cloth '*ndizvo*' is the right thing compared to the respirator. What are your views when comparing the respirator and the mutton clothe when exposed to mercury?

108R: Mutton cloth is used when there is no respirator. Respirator is what is needed. People use the mutton cloth because they won't be having the respirator. Respirator is also more expensive than the mutton cloth. You can also tear off a piece of cloth from any old clothes and you get a mutton cloth

F: Some miners say they won't use the respirator even if they were given they would throw the respirator elsewhere because it makes breathing difficult, what are your vies on the respirator?

109R_1: The respirator is difficult because you cannot share the same respirator and once used you throw it away.

110R_2: One of the challenges with the respirators is the poor quality of the Chinese respirators available at the market. It works for one day and breaks, yet it is expensive. Is there someone who has come across a good respirator?

111R_3: There is another good one which is comfortable.

F: Have you ever used a good one?

112R: I used it when I was at the large mine. It's not too tight on the nose like the ones produced by the Chinese. The Chinese respirator, small and too tight, you won't be able even to talk.

F: Ok, so the issue is with the type of the masks, there is another type of masks which work well.

113R: Yes, the good ones are also there.

F: I understand so for you, you are saying you are using towels. How do the towels compare with the mutton cloth? Which do you think is better?

114R: Mutton cloth is better because its long enough to tie, the towel is ready-made, you can struggle to tie some of the towels. The towel is unlike the mutton cloth which can be cut big enough for tying which makes the mutton work well.

F: So as you work, are you able to get the mutton cloth. How much does the mutton cloth cost?

115R_1: Some are $ 1.

116R$_2$: People move around selling mutton clothes**.**

F: Where do people move around selling mutton clothes?

117R$_1$: In residential areas in town.

118R$_2$: Even at TM mutton clothes are there.

F: So is it possible for you to get the mutton cloth?

119R: The challenge here is people don't get money in time. The doesn't come always. You can work for 2-3 months without getting money but working. You can be affected working like this when nothing is coming out. We need to get money to live well.

F: I understand, the next question is on your views on extracting gold without the use of mercury. Do you think it's possible?

120R$_1$: **I**t's not possible.

121R$_2$: How do you get the money without using mercury?

122R$_3$: The issue is our understanding is that mercury is used to extracting gold from the ore. However, if there is another method which make us work in better health we like it.

F: Any other opinion?

123R: No.

F: In the event that the other methods are not as efficient as amalgamation but help you work in good health. What do you choose?

124R$_1$: We chose amalgamation. We need to get all the money. After all this hard work one cannot afford to lose the money.

125R$_2$: We will rather stick to the old ways we were using before if the alternative methods are not effective.

F: What are the old ways you used to use?

126R: Whole amalgamation

F: I understand. I have heard that '*maheu*' is very helpful, and drinking '*maheu*' after dust exposure clears the dust. Have you ever heard of that?

127R: Normally, all miners get '*maheu*' after working. But the problem here is lack but '*maheu*' is encouraged. If you don't have '*maheu*' you can get scud especially for drillers because people affected most are drillers.

F: Is there something else which helps, some people say you drink '*muti*' to get strong. Is there something else like that?

128R: It varies among people but we know of drinking '*maheu*' and scud after drilling. Milk is not good because it has fat which can cause the dust to get stuck while dust scrubs and removes the dust.

F: But won't you get drunk and work while you are drunk.

129R: If you get drunk you can leave work.

F: In other places sickness are given different names like bone TB, are there any other local names for different sicknesses.

130R: There are no local names given.

F: How about beliefs on accidents, some miners don't use PPE because they believe that if 'something' (accident) wants to happen it still happens whether one uses PPE or not.

131R$_1$: That can be a belief among people but there should be order when people are at work. Anything can happen but one should go down organized, people must not have an attitude to say whatever happens, happens. Like work suits, it is important. If you do '*chiite ite*' (Haphazard operation), it's risky, any accident can happen and you get injured. We need to wear PPE and we need mutton cloth and we must wear. Dust will not stop affecting you because you are calling yourself '*Munija'.* Dust will still get into your system and overtime you will get affected. So it's important to work with protection. It's important to teach each other to protect not to destroy, protection is important.

132R$_2$: I think that person was expressing what is not right because of lack of money. Not using PPE is lack of enough money to buy PPE. One can choose to work with no helmet nor without buying milk to drink after dust exposure because he will be getting a sick salary. You can end up working without enough protective clothing but risk will still be coming to you.

F: Thank you very much. Thanks from your time and ideas. You may consider using what is available for now. Any questions?

133R: The issue is our country with the current situation, people say '*zvazvaita'* (whatever happens happens) because there is no money, and it's because of lack.

Things won't be organized at work and one will be considering that if I fail to provide at home, family members will die. Would you go and look for donors who could help small-space mining to get better and rise high so that artisanal miners live longer and mine longer. For now, we mine for 5-6 years and we die. This causes artisanal miners to die. Artisanal miners are dying. When I'm working underground, I open a small hole between me and this man with poor ventilation and I use explosives in that small hole. Despite fumes from blasting I immediately get into the small hole after blasting to get ore fast in order to get money to pay rent at home. Since you are outside the country look for donors for us so that we can get help for this suffering artisanal mining generation to live. So may you carry our message?

F: That is a good point. Is there another view. You are smiling, do you have another idea?

134R: No I'm thinking of going down the shaft to work. Maybe things are not well back home.

F: Thank you for your time. You may go and work.

Focus Group 3. Mine owners, Kadoma (2017)

F: Looking at small-scale mining, what could you consider as the health issues in small-scale mining?

135R$_1$: Water for washing and drinking. In old shafts the water can be contaminated with chemicals and acids from blasting fumes and acids. During work when one is hot or thirst one can drink or wash with contaminated water when thirsty and feeling hot

136R$_2$: Acid and mercury, amalgam burning. When burning amalgam there are vapors such as mercury vapor and one might not know the health impacts of the mercury vapor

137R$_3$: Thank you very much. When we consider all the health issues which one would you consider as the highest health risk exposure

F: Which one would you consider the most dangerous?

138R: It depends on how you handle the working situation but these are all dangerous but life should go on out of the dangerous. Everything is dangerous even not making money is dangerous even not making money is harmful

F: Thanks for your contributions. So when it comes to sicknesses and diseases are there local names given

139R: No there are no local names given.

F: I have heard of bone TB, have you ever heard about it?

140R: Yes there is bone TB, lung TB and back TB

F: So how is the bone TB?

141R: It affects the bone marrow

F: How about Lung TB?

142R: It affects the lungs; it can cause water in the lungs.

F: The back TB

143R: Same as bone marrow

F: Is the treatment different

144R$_1$: It's different

F: How about injuries, are there local names?

R:145 Rock falls, collapsing mines, breaking ropes. People can get injured or die. Then getting too much money harms people when they get over-excited and get involved in sexual indulgence after getting money resulting in contracting HIV, we have lost colleagues to HIV

146R: There is something I have forgotten which is faced in mines, insects such as mosquitoes get in the mines and bite people. Then rodents, rats come with ticks.

F: So miners are also bitten by mosquitoes and ticks

147R$_1$: Yes there are other places named Tick places where ticks are in an area with gold and people get attacked and injured by ticks

148R$_2$: There is also the challenge with 'Bember' (slasher) many people who raid and attack miners and get other people's ores. They can siege mine owners to injure and raid them. There are many cases like that.

F: Thank you, how easy is accessing health care as an artisanal miner

149R: It depends on whether you are injured or not. If injured, the police report is required. I don't know what they do with people with no police report; maybe they bribe and get medical attention. Any other ordinary cases are treated like any other ordinary citizen

F: On payment, do you always pay or there are things you do not pay for

150R: With TB you do not pay. You will be treated as government property but at the beginning. You pay until diagnosed TB positive then you will be declared a government property

F: There is an Occupational Health Center being established at the hospital. The center seems to aim to focus on issues like occupational injuries.

151R: Yes they are trying to focus on that especially for the injured unregistered artisanal miners who normally receive delayed attention due to the layer of pre-requisites they are expected to fulfill before they get medical attention. With the Occupational Health Centre such cases could receive timely medical attention.

F: Do also you pay for malaria?

152R: Yes

F: I understand. We also got the information that malaria, TB HIV are treated for free from Kadoma Hospital. Is it like that?

153R$_1$: When you get to hospital you pay to get the card and the tests, it is free after diagnosed of malaria, if the hospital has run out of drugs you may be asked to buy medicine.

F: Do you also buy TB medicine?

154R$_1$: When the hospital has run out of free TB drugs you go and buy.

155R$_2$: Another point I forgot when I was explaining public health problems in artisanal mining, people get bitten by snakes, snakes hide in timbering.

F: Is snake bites serious?

156R: Yes, yes, yes, we have had serious cases where people get bitten by snakes and die. There is also the problem of scorpion bites.

F: There was mention of outreaches at the hospital, how is it helping in the artisanal mining communities.

157R: Full coverage might be lacking but in other places health workers visit on a bicycles.

F: The health care professionals expressed that turn up is very low when they visit the artisanal mining communities. May be they need guidance on places to get the artisanal miners

158R: I use to collaborate with the Kadoma hospital outreach team, when they were distributing mosquito nets to the miners because miners sleep in the open.

F: How about these days, the health care professionals mentioned that regular outreaches to artisanal mining communities are still ongoing.

159R: They should have given specific reference to the areas they have been visiting; we haven't seen them recently in the artisanal mining communities.

F: What do you think should be done for miners to get medical attention at the hospital?

160R: Free services.

F: Are there artisanal miners who completely fail to access health care.

161R$_1$. Prompt medical attention for every citizen in need of medical care

162R$_2$: The majority fails to access health care even to the point of death because they cannot pay consultation fee at the hospital.

163R$_3$: If a mine collapses it is difficult to get help, colleagues can run away because of the rear of the police. If the case is reported the police will ask for a statement so the fellow miners keep quiet. The incident may go unreported yet someone could be rescued and saved. The miner's association should explain to the police that we are surviving from artisanal mining and when an accident has happened its happened because people are hungry they need money so in case of accident post must not just ask for explanation, helping should be prioritized and at the hospital, help must be given before police reference. I n the event that someone is bleeding heavily urgent attention should be given before police reference.

F: So besides going to the hospital what else is done in the community to help the sick?

164R: It depends on relationships and people around the sick person. If someone had poor relations like raiding mines, if they get sick they can be abandoned. Foe me I won't help a raider I will rather increase pain. But in general people in Zimbabwe have a kind heart to help. It's troublesome people who might be abandoned.

F: What else can be done to protect health in artisanal mining?

165R$_1$: If we could get clean water, it will protect health. The available water can be harmful to water. People must not come across water and drink; water must be taken to the laboratory for testing.

166R$_2$: In mining there must be avenues to get prompt results like after taking water to the laboratory for analysis. Many questions and many charges will make people look for survival methods without taking the water for testing. People can look for short-cuts which can result in harmful effects in the future.

167R$_3$: Shafts must be made safe. People must use cement for shattering but the costs are high especially at the beginning and people can resolve to use timber. Trees for timbering are getting finished in the forest. Some types of trees which used to be there in the past cannot be found in the forest or are now in distant forest and is costly to fetch. Most people therefore work with no timbering resulting in collapsing mines.

168R$_4$: Timbering also causes high carbon monoxide which is exacerbated by blasting.

169R$_5$: Most of the shafts have poor ventilation which is below the expected standards. Things need to be standard.

170R$_6$: That's why some miners say they cannot wear protective clothing; the problem is limited working space and poor ventilation. In the standard shafts people can work with protective clothing.

171R$_7$: Good ventilation is expensive to achieve. We do collective bargaining people cannot work for developing the mine; they want to mine where there is gold. If shafts were to be sunk properly, 2 ventilation shafts are needed for good ventilation.

172$_8$: The mining methods we are using are for survival tactics without t proper min9ng which cause unsafe working conditions.

173R$_9$: The other challenge is if we g o to the bank to seek a loan to develop the mine, the bank requires title deeds. ASM is considered a risky business, but ASM is supplying foreign currency to the country.

F: How do you view the issue of formalizing ASM.

174R: According to what I think once one gets the mining registration and certificate that is formalization. Do I need to register a company what for? After registering the mine and following procedures I should be recognized.

F: It has also been raised that there are some artisanal miners who are not known who move around digging, are there many miners with mining certificates?

$175R_1$: Yes, they are there.

$176R_2$: 95% of the members of the miners' association are working in a registered mine. I have even challenged the police to show me mines who are digging with no rights, and they have failed. Most miners are registered; those who are not registered could be 5%.

$177R_3$: The other issue with registered mines is management. We work with artisanal miners who want to work independently even when employed.

F: There is another issue that miners could use PPE. What are your views?

178R: PPE, is good, the helmet protects from stone injuries.

F: The challenge is who will provide PPE, the mine owner or the miner.

179R: We work with shares. For me I have capital to sponsor the mine but both the mine owner and the miner must contribute to buying PPE. The challenge is if the mine owner provides PPE, the new miner can disappear in 2 hours and you buy again for the next employee.

F: The challenge faced by mine owners who are providing PPE is that the employees can wear as they go down but take it off when they are underground. SO what do you think mine owners to ensure that PPE is used underground?

$180R_1$: Ventilation needs capital, mine development needs capital. Capital should be made available to miners after getting the license.

$181R_2$: Miners should also be challenged develop interest in using PPE.

$182R_3$: The other challenge is security, there is need for security to avoid raiding but security is very expensive. I have just received a bill of 2 350 USD which has been accumulating

F: The other challenge is some artisanal miners were saying they won't use PPE even when they get it. Who is going to monitor and make sure PPE is used consistently?

183R: There should be mine regulations for PPE to be checked at entry and also underground. Safety management should be available to monitor use of PPE because if not monitored it's still a cost when a worker gets injured.

F: There is another major issue on mercury use. I n the beginning it was mentioned that everything is risk even not making money is risky. Do you think it is possible to reduce/eliminate mercury use in artisanal mining?

184R: I f you come up with another technology and demonstrate the gold recovery %we could opt for that because mercury is also a cost to us.

F: There is also the issue of retorts. Where we visited most mines had retorts but we also learnt that the retorts were just for inspection not for use.

185R: When it was introduced we did not take it up. But it's a good thing, it recycles mercury and we keep using the same mercury. The problem is we are used to our survival technics.

F: Is the retort available, is it cheap? I f its expensive people prioritize affordable methods. However, if the retort is cheap and readily available we use it.

186R: The retort can be made cheaply with affordable material worth 10usd. We can do it next week, I can come and demonstrate.

F: Some mines expressed that the retort takes more time than open amalgam burning. Would you still go from the retort given the fact that the retort still gives high recovery, recycles mercury and protects health?

187R: We will get behind time because our job in artisanal mining is seasonal; when rain begins we focus more on farming. That's why people opt to pay for transport to get to town instead of walking to gain time. If the retort is slow we will put it aside, if the retort is time efficient then we will use it.

F: How about doing other mining jobs while the retort is processing the gold.

188R: You cannot leave gold processing; it is not possible*. (Hazviite kusiya staff*).

F: Mr. X what can be done to improve the speed of the retort.

189R: You can use gas to make it faster.

F: What do think about using respirators when burning amalgam. There is the SO2 respirator which is being reported by other miners as efficient. You can buy filters and replace and it costs 40 USD.

190R: If affordable we can use, there used to be to be another respirator with replaceable filters but the filters were expensive. Though it was good, the same mask could be shared while changing filters.

F: Mine owners could be more affected by amalgam burning because they are always involved in amalgam burning.

191R_1: Everyone is involved; all the miners will be there during amalgam burning.

192R_2: But mine owners are affected more. Say there are 10 groups, the mine owner will be there for the 10 groups. The mine owner could be involved in amalgamation every day.

F: If you look at it again, mine owners make money and buy things like cars, houses, livestock. Could mine owners be able to also buy respirators.

193R: If you are in artisanal mining you can die anytime, so things should be done fast before one dies.

F: What do others say?

194R_1: It's good to protect health.

195R_2: We must try to keep healthy as our children are still growing, if we die early our kids will become street kids.

F: Have you ever heard of the Minamata Convention, the UN IS proposing reducing or eliminating mercury. What are your views on this proposal?

196R_1: We don't like mercury, we forced by situations. If there are new methods that make it possible for us to extract gold without mercury, we can stop using mercury. It's also these organizations which brought mercury.

197R_2: We only need a friendly and efficient method of extracting gold.

F: How about medical checkups, what do you think about medical checkups?

198R: The problem is when you get to the hospital and you are told you have a medical problem you get sick because of stress. Yet what you don't know doesn't kill you

F: What do others think?

199 R: Checkups are good, the problem is money, consultation fees, x-rays, and blood tests are expensive.

F: I f the will is there, eventually it will happen.

200R_1: Willingness is there, what is needed is money.

201R_2: The whole process of seeing the doctor is expensive.

202R_3: Health must be covered by government policy. Health should be made accessible. We normally go to the hospital when something has gone wrong, not for medical check-ups when one isn't sick.

203R_3: People might be working with mercury getting affected without knowing. We need TV adverts on the health impacts of mercury so that people know. Just like tobacco, it is well advertised on TV people can die from tobacco smoking but they know the impacts of tobacco smoking when they are smoking.

F: How about making use of the hospital outreaches? The hospital reported poor turn up in mining communities during their outreaches.

204R_1: The problem is the hospital staff cannot go alone to the mining communities, they need to involve members of the artisanal mining federation. If they go alone, they may face resistance.

2005R_2: They must give supper; some people might even donate blood.

F: Thank you very much for your contributions

2020 FGD

Man miners Shurugwi

What do you understand by safety first R

R1In mining, safety first means protecting you before engaging in work activities.

R2Safety is in all everyday activities, so safety is essential in life.

F: Which other safety measures do you have in mining?

R1 Wearing PPE

Which accidents have you experienced?

R1 Safety is not only in PPE; it's even in how you talk and interact with others at work. When you speak in ways that can annoy someone, that is not safe. You may provoke a reaction leading to violence, fighting, or stabbing.

R2 There are issues like failing to understand each other because of money. Typically where there is money, there is fighting, violence, attacks, and stabbing. Attacking and stabbing someone is not safe.

F: What are the other safety issues?

R1 Mines involving more extensive operations need security and writing down the names of people working in a specific shaft because we have lots of issues. When listed people get into a shaft, there are cases of raidings that can involve injuries for the people working in the shaft, '*Mashurugwi' (People who come from Shurugwi).* For us, we are listed; perpetrators invade because there is no security to protect the workers.

F: Thank you very much for the information. What else could be done by the workers to protect their health?

R1 Wearing a mask

R2 Verbal warning before blasting, shouting 'Explosive!!!!' and people move 100m away.

R3 After blasting, allow 1.5 hours for the fumes to clear.

F: Thank you very much. How do you burn amalgam?

R1 This is the area we need to know how to protect ourselves. For us, we just burn in the open when we are bare like this. We just get our amalgam and burn without protection. We know the dangers of working with mercury and acids, but we do not have the means to protect ourselves.

F: Is there someone who knows how to protect you from mercury, acid vapors

R1: Knowing the wind direction and burning while you are in the opposite wind direction so that you inhale less mercury and acid vapors. When burning amalgam, you must not blow with your mouth. You must use a plate when you are in the opposite direction of the mercury vapor to reduce mercury intoxication. The health impacts of mercury are chronic

R2: You can use a cutting torch

R3: Acid must not be in contact with the skin; it can damage the skin, especially before dilution

F: Have you ever heard of a retort?

R1: No, we do not know

R2: The challenge with the retort is you get less and purer gold sold at a low rate for gold with some impurities

F: How about mercury-free technologies?

R1: Alluvial gold is processed through panning without mercury. Reef gold is processed with mercury. James' table is mercury-free. There is also a tree that can be used to detect areas rich in gold, seeing gold.

F: When you get mercury-free technology. Do you think people will use it?

R1: It's not possible to get gold without using mercury

R2: As long as there are no extra costs, it could be accepted. The problem is ASM miners calculate the expenses from digging to processing. One can lose money in the supply chain. So if the new technology does not involve extra costs, it is good to get the gold without using mercury.

F: For others, why do you think it is not possible to use mercury-free technology?

R1: Because mercury is the only method to get gold without losing.

F: Do you think your family can be affected by mercury use?

R1: They can start shivering.

F: Where do you keep your mercury?

R1: In the house

R2: In the pocket

R3: Anywhere you will not lose it

F: So where can we keep the mercury?

R1: At the mine

F: Do you experience any other safety issues?

R1: Divorce

F: Could you explain more about that?

R1: When a fellow miner who is single gets more money than you. Then, your wife can be attracted and leave you for the fellow miner.

R3: Mining with women is difficult. Some women will be looking for money in exchange for favors (sex)

R4: STI's like syphilis**.** There are no free condoms. Sex is without protection.

R5: There are few women in mines compared to men, which can cause fighting among men even violence.

R6: Challenges with visiting our families because of erratic salary

R7: Some men get involved with women they meet when mining and stop visiting their families.

R8: Some women go to mining areas to look for money from men

R9: Some neighboring men n ASM get money more frequently and visit their families more regularly. And get involved with the miners' wives who rarely visit home, affecting relationships between spouses.

F: How do we take care of equipment and tools?

R1: After working, we pick up and gather our tools

F: How about safe drinking water underground?

R1: That one is a challenge

R2: For us, , if we know the situation in the mine. We get portable water and go down with portable water and go down with drinking water.

F: You are doing well. What are others doing?

R1: For us, we drink the mine water underground

R2: If the water is clear, we think the water pollutants can be in the water, so mine water might not be clean. But the mine water is what we have been drinking.

F: What are the issues with drinking where you are mining?

R1: When we need the 'toilet, 'we also relieve ourselves underground where we mine

R2: The issue is thirst has no timetable. One can get thirsty at any time, especially when one is working. So when you are working, and you are dehydrated, you do not think of health issues, you think of quenching the thirst and going back to work. So when we see clear water and do not get immediate effects after drinking, we assume all is well. So when working underground, we drink the water underground.

F: So, how is the water in the shaft?

R1: Noise and hamming. There are also explosive fumes.

F: So how is going down with water when mining underground?

R1: At our mine, water is pumped from the shaft where we work. The water passes through contaminated areas since people relieve themselves underground when they need the toilet.

R2: Even borehole water is contaminated because boreholes are open. Some people throw their waste into open boreholes after their activities. You can get used condoms and women's items when getting water.

F: How about boiling the water?

R1: The problem is with time. Time is money. If you lose 2 minutes of boiling water, you have lost money.

F: Can you boil the water before you sleep?

R1: The problem is we overwork. After working, we get over-tired. So lose attention to boiling water. We also need to meet a target.

F: What challenges do you have with safety and health in your work?

R1: Equipment

R2: Difficult to follow safety rules because some things require you to put money when looking for cash. So you can end up saying '*chero zvazvaita' (Whatever happens)* so that you get money to take care of the family instead of working for health and safety impacts after getting money.

When you get a good site, someone will come with ownership papers and take over the area.

R2: The name 'chikorokoza' [informal] is associated with no regulations. No one can give rules to another. People work and go. There is a need to transform the name. Miners should prioritize safety and security systems after getting money.

R3: Food safety. Women who were cooking were serving GMO products, especially chicken. The meat was not well cooked. Soda was being added to the meat as a meat tenderizer. ,

R4: Need for worker representatives for ASM

R5: Raidings at milling centers

Focus group discussions women Shurugwi

F: Thank you very much for participating. Have you ever heard of safety first?

R1: Yes, we have heard of protecting yourself

F: When doing your work of washing. How could you protect yourself?

R1: Gloves

R1: Gloves

R1: Gloves

F: Some participants have expressed challenges with getting the gloves. For you are you getting the gloves?

R1: Gloves are found. We bought. The problem is mercury and sand gets stuck in the gloves and the nails. The fingers will be exposed to concentrated mercury in the gloves, so we find it better to work with bare hands. We tried it, and we have the gloves.

R2: There is some mercury which was new and not tested we used at the other site I once worked. All the women who used that mercury got unexplained weakness and sickness instantly after using that mercury, and we stopped working at that site.

F: Did you manage to access the clinic during that sickness

R1: Yes I went to the clinic, and I was given some tablets.

F: How about you, what is your experience

R1: Yes, when the mercury gets into the gloves. It stays there. We bought the gloves, and we could not continue using the gloves.

F: What safety issues do you face issues like accidents?

R1: We do not get accidents, but we are left alone with no support. Like now, we do not have jobs. When we go to the mountain to search for gold, we get good sites. Once we open and get the gold, you cannot work there. Men in authority come to push us out, work and command us not to come back.

F: Are you saying the men do not want women to work?

R1: They do not want everyone found on site, even the men. So far, we have opened two good places X and Y, which were taken away from us.

R2: Even now, we have less work; the mills have less ore because the good mines were taken away.

R3: They come, take away the ore and declare that they do not want to see anyone on the site and get the mining certificates.

R4: For us, we do not know where to go and report in that situation.

R5: It's even difficult to get jobs because there is less ore to process, which causes hunger. We have children who must go to school, and the children need to eat. At home, because there is no food, we always have conflicts with our spouses. So we do not know what to do. We have nowhere to work.

R6: Active mining areas have been taken away, and there is nowhere to mine

R7: These are the problems. We open up the area, and some people take the site(s) away from us. We open after opening up; we are moved out. For us, we do not have the money to register. We go out to search for gold. When we open up, we are chased away.

R8: Women open up. After opening up, they are chased away, and they cannot work there.

R9: The person who takes over, registers, brings his security. Then, the security selects their relatives to continue working, and the rest are chased away.

F: Oh, I understand; the founder of the ASM association is with us. He will discuss these issues with you. Are there other issues affecting women>

R1: This is the main issue. Now we are afraid to look for money, and we die of hunger because we have nowhere to work.

F: In other places we visited, women are not allowed at the shaft. Here are women allowed to get to the shaft.

R1: The challenge is for us to get into underground shafts; we cannot because we are women

R2: For us, we do alluvial mining; we cannot mine underground. We wait for our children and husbands to work, but now our children and husbands are not allowed when we open up.

F: After getting the amalgam, are you involved in burning the amalgam?

R1: The gold buyers burn for themselves.

R2: For us, we do not burn the amalgam

F: Do you watch the gold buyer burning?

R1: For us, they burn the amalgam and treat while we wait and watch until they weigh.

F: Do you protect yourself from mercury while watching?

R1: We do not smell the mercury. The mercury affects the person who blows.

F: Mercury can affect people up to a 2 km radius. Do you think you need respirators for protection while waiting?

R1: For us, we do not always burn amalgam. The owners of the ore pay us. We remain behind while the owners go to the gold buyers, where they burn, sell, come back and pay us.

F: There is a program on mercury-free technology. Do you think you can get gold without using mercury?

R1: No

F: Why do you think like that?

R1: What we know is we use mercury to get the gold. The gold that does not need mercury is alluvial.

F: What if a new technology is introduced?

R1: Does the new technology involve machines?

F: Yes

R1: That will disrupt us because we can die because of poverty. For us to get the money we wash for those using mercury. That is how we work to get paid. So if you bring machines, where do we work?

R2: We do not go down the shaft for us, so we can wash for those using mercury.

R3: The Chinese brought the one that is not good for us, it washes own.

R5: Since we cannot mine, we get a job to wash with mercury for the men with ore. That is our job which is making our families survive. So if they bring machines, they disrupt us.

F: Can you organize yourself, get machines, get people to work, and manage your site with equipment?

R1: We can organize ourselves, but all those things require money. For us, we do not have the money.

F: What if there is a program for women?

R1: That will be good. The problem is when we open a place; we get chased away before getting machinery.

R2: For us, we do not have mills. When we bring our ore for milling or to the gold buyer, the news of our gold spreads around. We cannot work for two days, the next day, you find the place taken away, and you can be beaten and chased away and can slip-sliding down the mountain. The invader will process papers and get the mining certificates. When the security from the registered owner selects people to keep working, they choose their relatives.

R3: Some women have found good places with gold and have decided not to disclose their areas

F: What are the other issues you are facing as women? Some men raised that working with women is problematic because women want men's money; a; a woman can attract you to give them all the money without feeling the pain of losing money?

R1: That is not for all women. Some women come for that. For us, we come to work. We do not have time to waste following man. We move from site to site. We are working for our families. We can earn more than spending time following men.

The founder of the association joined the discussion and explained that there was protection for women. The women could report to the ASM association, and investigations could be conducted such

that the registered miner will agree for women to work on the site they opened. However, membership was required, 15 USD, to get such support.

Women married to miners Shurugwi (2020)

Thank you all for participating. What is your opinion on the involvement of children under 18 in mining?

R1:I think it's not good, children will start smoking, and once children get money, they lose focus on going to school. The children will begin focusing on getting more money, getting a few points to buy alcohol and cigarettes. Taking drugs like marijuana is common in ASM. So the child will change the way of life and be disrupted once the child starts getting involved in ASGM. Once children start working in ASM, they end up not going to school.

F: Any other views

On respect, the child ends up disrespectful, speaking vulgar words. In ASM, they say vulgar words; nothing is unacceptable.

F: Thanks, any other views

R1: It depends on the background of the child. Suppose the child is orphaned, staying with a relative who is not paying his fees or staying with a step farther not willing to pay fees for the child. The child might decide to mine to get money for school fees. So there are different situations. Such a child may work and go to school rather than staying home without going to school.

F: Do you have any programs that support disadvantaged children with school fees?

R1: Not known in the compounds

F: Do you have cases of mercury contamination affecting unborn babies and child development?

R1: No such cases, just heard that mercury is poisonous

F: Do you know of challenges faced by women in ASM?

R1: The shafts are too deep for women. Women are not able to work in shafts.

F: Are women willing to join mining?

R1: We are willing. There are limited places for mining. Some areas no longer have gold.

F: Have you ever-mined?

R1: I once mined. For those who want to start, they can be told there no new areas to peg.

F: Is it possible for women to get their claims?

R1: Who will stand for women to get their claims?

F: Is it something women are interested in?

R1: Yes. The challenge is men usually pull women down. We can be despised, yet we have desires and wishes to do something as women, and there is no one leading us to get there.

F: Some women are saying once they get the gold, they are chased away. Have you ever heard of that?

R1: That is common

F: What are the other issues faced by women? Some women were talking of rape cases. Have you ever heard of rape cases?

R1: We haven't heard of such cases.

R2: If you have money. You can be raided.

R3: In other places, women can be raped when coming from work, when walking alone.

F: From your experience, can you say sex can be affected by your husband's work, like the use of mercury?

R1: Mercury has long-term impacts. People can start reacting without knowing. It will be good if people could check. People can be poisoned not knowing. There is a need for a program to test mercury in ASGM.

R2: The other thing is tiredness, long working hours, and heavy lifting affecting sex.

R3: For me, when I used to work. I used to get so tired that I would get home and sleep because of overworking.

F: Do you think it's possible to take breaks in mining?

R1: Breaks and seasonal mining is associated with severe hunger since the money from mining is hand to mouth. If there is no mining in the rainy season, children will die of hunger, and there will be no school fees, murder, and stealing will be on the rise.

F: Do women married to ASM look for other men when their husbands are not earning from ASM

R1: That is possible but for some women that is waste of time. Some women always hope to have other men. It depends on people. Some women are satisfied with what they have. Such things have nothing to do with ASM

F: Do you have cases of children who secretly join mining without permission from their parents?

R1: No

F: Are accidents which involve loss of husbands common

R1: This side it's not common. It's common in Wonderer where a mine can just collapse when people are underground 15, 30 or men, many women and children lose husbands and fathers

F: What happens to the children and women?

R1: No compensation. ASM is self-employment.

F: Do the widowed women join mining?

R1: Some women join and wash. Others look for alternative ways of livelihood such as trading

F: Safety, health and protection. What can be done for safety, health and protection?

R1: They have PPE, they wear PPE but where they work underground is dangerous. PPE cannot stop mine collapses. Like these days it's raining, someone can be underground wearing PPE and the mine collapses. The issue of PPE started long back, most men leave their homes wearing PPE. However a person cannot be protected by PPE against a heavy rock fall.

F: In your view, who should pay for safety, the sponsor or mine owner?

R1: Mine owners

R2: The miners go underground. Miners should timber under guidance and supervision. So it is everyone's responsibility.

R3: Associations must make follow up. In rain season there are many accidents. There is too much water now and shafts are weak.

R2: When I was mining, I was encouraging people to check safety before going down.

Facilitator: Thank you very much for your time.
