# Supplementary material for: Hazards and Control Measures among Artisanal and Small-Scale Gold Miners in Zimbabwe
Source: Ann Glob Health. 2022 Mar 15;88(1):21. doi: 10.5334/aogh.3621 (PMC8932352; doi:10.5334/aogh.3621)
Supplement: Data set. — 20202 Health and Safety Survey among Artisanal and Small-Scale Gold miners in Kadoma and Shurugwi, Zimbabwe. [file agh-88-1-3621-s2.zip › s2-agh-3621_singo/Data_Set_4_IDI_SummaryNotes_Dat.pdf]

**Describe your usual working day**	**What are your health needs at work**	**Describe what could be dangerous to your health at your workplace**	**Which PPE do you have at your workplace**	**Would you use PPE if you had a choice**	**What are the challenges with using PPE**	**What is the impact of mining on your health and your daily life**	**Possible health interventions**
1.Mine Owner **70 male**<br>-managing the hammer mill and the mine<br>-housekeeping at the mine(cleaning, checking water, taking out the dump)<br>-amalgamation Crushing(wet crushing). Blasting was done using dust masks. Lashing followed 4-5 hours after blasting or 2 hours after blasting by compressor. There was 1 TB case of treated TB at the mine, treatment was paid for by the owner.<br>-Accidents at the mine were caused by negligence . –It was expensive for the miner to change the rope which was used to carry the miners down to the shaft and up every 2-3 weeks.<br>-mine owner preferred metal ropes which could last 2-3 months but he could not afford the cost.<br>-The mine owner mentioned a case of a broken leg which was caused by using a rotten robe for carrying the mines down to the shaft and up to the ground. The guy was taken to the hospital by the mine owner, he recovered and was limping. . The employee stopped working in the mine and was given capital by the mine owner to start a mini-grocery shop to earn a living<br>-The miner owner indicated that registration for	-local health center. The mine owner reported that people were always sick; stomach ache, headache, influenza, pregnancy, HIV & AIDS, stroke.<br>It was reported that people could die without seeking health care<br>-Delayed health seeking was caused by transport challenges to go to the referral hospital<br>-The mine owner referad to cholera outbreaks at the mine compound in 2008 due to poor sanitation and a treatment shelter was established at the compound under the administratio n of a healthcare professional	-Collapsing mines due to opening cracks<br>-'It's always dangerous'.<br>-The mine owner explained that the foreman must check the condition of the mine before working and the need for the mining workers to be cautious<br>-The miner owner expressed that mercury was a silent killer since it was chronic<br>-Noise was coming from hammer mill, generator and blasting. Blasting was described as 'shaking' the houses in the homestead which was located within the mining site.<br>- The mine owner commented that many people die from mine accidents and collapsing mines in informal, individual and rushed gold mining, there was the mentioning of a guy who got into the shaft alone, was closed and died<br>- It was mentioned that there were incidences of mining in small holes where people could be closed in, in rainy season. It was explained that most of such cases in informal mining were realized later	-gloves<br>-masks (same masks for mercury, and dust)<br>-respirators for nitric acid<br>-goggles<br>-waist belt tied on the same rope with the bucket for carrying the miners down to the shaft and up to the ground	-yes	-Workers had the tendency of not using PPE and taking it and leaving it home where items such as the mining gumboots could be used for farming<br>-The mine manager was making the workers who leave there PPE at home pay for replacement<br>-negligence of not putting on the helmets<br>- It was reported that the workers normally use PPE when they are new<br>-Most of the workers were not wearing their PPE despite having received PPE from the mine owner<br>Observation<br>The miner's narrative revealed that teaching and training on the mine was more focused on technical mining, not on PPE	-Infections in old age<br>Observation<br>The mine owner had cars, a decent homestead, and researchers were given cold soft drinks more than once and potato crisps	-Interventions addressing TB, injuries, pains, sicknesses<br>-PPE<br>-A local clinic. The mine owner expressed that there was positive-health seeking attitude in the community

National Socal Security Authority (NASSA) which gives security insurance and compensation for occupational injuries was not possible for informal small-scale miners, NSSA membership is normally for civil servants<br>-Discussion with the leader of the Zimbabwe Artisanal and Small-Scale Miners Association ZASMA revealed that ZASMA was working towards NASSA registration for artisanal miners.							
2.gold buyer, supervising hammer mill, burning amalgam **39 male**<br>-working on commission	PPE: gloves, gumboots, work suits, dust masks and respirators (masks and respirators were not available)	Burning amalgam	gloves, gumboots, work suits	Yes. Depending on the day	Hot weather	-tiredness<br>-Injuries had an injury on a finger when he was fixing the hammer- mill belt. 'I had gloves but I was not wearing them'<br>NB*No penalty was given to this miner by the management which gives PPE	-medicines for common ailments
**3.**mill operator **male 27**	Dust<br>Smoke from the mill<br>Respirator<br>Gloves<br>Gumboots<br>Work suit	Smoke from the diesel engine	Gloves<br>gumboots	Yes<br>But was not wearing the PPE given by the mine owner	Difficult to use because of hot weather	Not so much	'Don't know
**4.** Cynadation.<br>**Male 29**<br>- 'It was commented that cyanide was used to commit suicide (sourced and used outside mining activities)	Respirators	'Cynadation affects through saliva and the digestive system'<br>-not washing hands 'no food during working hours', 'wash hands with fresh water before you eat<br>-	Gumboots<br>Respirators<br>Work suits	Have to use<br>'Sometimes forget gloves'	Circumstances such as stress and oppression cause lack of use of PPE	'A lot'<br>TB (The client had no coughing or chest problems)	'Leave to the boss'(confidential)
**5.**marketing gold, financial management, procurement, amalgamation **male 50**	Need to be taught	Safety<br>Protective clothing<br>Untrained blasters	Nothing	Yes<br>Consistentl y	The miners think everything is alright	-Lungs TB<br>-Eye-sight when checking the level of gold purification with the cutting touch during amalgam burning<br>-need for respirators	education
6.-Gold detecting<br>-Mining: checking machinery,	Drinking milk after blasting to clear	Safety<br>Fumes from explosives	Gumboots<br>Respirators<br>Work suits	No	Limited understanding of English	Limited understanding of English	Limited understanding of English

procurement, explosives<br>-blasting **male 33**	blasting fumes and dust	Injuries from drilling	Eye protectors<br>Gloves<br>Gumboots<br>Helmets<br>Waist belt				
<u>7</u>.blasting<br>-Lashing (moving ore up from the shaft)<br>-Delivering ore for milling<br>-amalgamation with bare hands<br>Blowing (using the mouth) during amalgam burning<br><br>**Male 36**	-underground respirators to protect against blasting fumes (after waiting for 3-4 hours) and mercury vapour when burning amalgam<br>-gloves<br>-unavailability of PPE	-Inexperienced blasters<br>-first AID kit and first AID training for blusters (participant had lost a brother, the previous 2 weeks, the body was found in the shaft, there was no suspected rock-fall or electric shock).<br>-poor timbering<br>-lack of PPE<br>-Lack of training of miners	Gumboots<br>Work suits<br>Respirators	Never (participant was drunk)	No response(participant was drunk)	-Inhaling carbon<br>-electric shocks<br>-lack of experienced electricians	-Precautionary measurements<br>-Education
<u>8.</u> mining<br>Drilling<br>blasting<br><br>**female 31**	-Safety precautions: no safety talks, things have changed, more gold rushes<br>-ignorance on the effects of mercury<br>-ear morphs: high levels of noise from blasting and drilling	-accidents can cause death when blasting<br>NB*Thought burning amalgam was not dangerous.	Gumboots<br>Helmets<br>Work suits	Sometimes<br><br>Could go to work in ordinary clothes	Gold rushes, some miners feel like they are losing time by getting PPE when leaving for a gold rush	TB 'of the bones' (was taking milk after blasting and mining to clear blasting fumes and dust	Education
<u>9.</u>mining<br><br>**Male 33**	PPE: helmets<br><br>Gumboots<br>Work suits	Fumes from blasting	Helmet<br>Gumboots<br>Work suits	'I must use PPE'<br><br>'Using every time if available'	Different ways of working	No known impact (participant was working in his own mine, digging in a wet shaft)	Information giving
<u>10</u>. mining<br>Amalgamation<br>Blasting<br><br>**Male 43**	Helmet<br>Gloves<br>Eye protectors	-Blasting fumes<br>-Accidents (not too much)	Gumboots<br>Helmets<br>Work suits	Commented could use (question 2)	-Availability of masks and respirators<br>-The cash flow in ASM is fluctuating, informal miners can be able to buy at certain periods and no potential to buy when they have no money	-Enough things<br>-Buying enough food	-
<u>11.</u>Mining after hiring blasters<br>Amalgamation<br>Amalgam burning<br><br>**Male 32**	-safety<br>- protection from inhaling mercury	-TB because of blasting with no PPE<br>-accidents and loss of life due to lack of skilled blasters<br>-Safety due to mining of pillars	Nothing	Yes	-Lack of knowledge<br>-Most miners are new and are not used to using PPE<br>-Pride	No impact	-giving PPE<br>-Programs on safety
<u>12.</u>Gangleader<br><br>**Male 26**	Respirators<br>Helmet<br>Gloves	Rock falls<br>Blasting fumes<br>Dry blasting	Helmets<br>Gumboots<br>work suits	Yes<br>Could use all-times or	-ignorance<br>-PPE can be expensive for	-Chest problems<br>-eye problems<br>-loss of ability to	Use of PPE

	Safety gumboots	Slipping<br>Breaking ropes		sometimes	individuals<br>- Hot weather for work suits<br>-gloves are difficult to use with jag hammer and shovels<br>-gloves can catch some gold<br>-It is not possible to use gloves when panning, the gloves may break, sponsor will not like losing gold<br>-Gumboots can be too hot and cause heat blisters	work	
**13.-**Mining after drilling, use of the rope for going down the shaft and up to the ground<br>-Supplying mining equipment and explosives<br>-milling<br>-amalgamation<br>**Male 29**	-Upgrading winch pullies to machines<br>-bigger pits<br>-limited capital	-Lack of PPE use<br>-manual operations<br>-lack of instruments to test levels of gases when blasting and levels of noise when drilling	No PPE and no regulations	Yes. 'It's wise because there is accidents'<br><br>Always	-It's difficult<br>-It takes time to teach people<br>-Sub-standard shafts and small pits<br>-Too much heat underground<br>-discomfort when wearing helmets<br>-Though PPE is available and not too expensive respirators are disposable and difficult to replace, unsustainable<br>-Pple use maheu (local liquid food) forclearing dust and fumes	-Livelihood source<br>-acguiring property cars and porperties<br>- Miners don't go for medical check-ups which affects their health	-Continual educational workshops on safety and PPE
**14.**Managemant, supervising amalgamation<br><br>**Male 55**	Maheu (local liquid food) to clear dust	- Air pollution from amalgamation and dust<br>-noise from jag hammer	Helmets<br>Work suits	Yes<br><br>Every time	-Mining should be done according to regulation<br>-People need lessons to mine properly and move away from informal mining	-Source of livelihood<br>-The participant reorted that he was going for medical check-ups monthly and commented that some miners are afraid to check for check-ups because of the fear of HIV	-Workshops on safety and use of PPE down to top
**15.** Mining after blasting. Used manual winch for going down. Mentioned occurrences of injuries when going down, falling mines and the challenges associated with fumes from blasting<br><br>**Male 42**	PPE to protect the mouth and the feet(from water)	yes	Carbon from blasting fumes	-work suits<br>-safety shoes	-PPE wears out before one gets money for replacement<br>-difficult to buy for oneself<br>-preference to use PPE for other purposes, participant was wearing a goog work suit at the shops<br>-1 pair is not available after washing	-Life can turn out for the better through quick money when there are no accidents experienced<br>-Long term health impacts from blasting fumes	-Education on safe mining and use of PPE<br>-Regulations to enforce use of PPE from mine owners. It was mentioned that most people work under someone

					-saving the worksuit so that it lasts, worksuit gets worn out in 3-6 months		
**16.**Mining after blasting: dry mining going down by rope. There was noise at the mine<br>**Male 29**	unknown	Carbon from blasting fumes	-gumboots<br>-work suits respirators<br>-helmet<br>-gloves<br>-work suits (no dust masks, no ear plugs)	-use<br><br>-sometimes	People not used to working with PPE	-No known health impacts Participant reported that he had always used PPE	Not sure
**17** Detecting gold, amalgamation<br><br>**Male 48**	Gumboots<br>Gloves<br>Masks<br>Goggles<br>Work suit	-Using a cutting touch without goggles<br>-amalgamation	-gumboots<br>-work suits respirators	Yes Consistentl y	-Heat and limited working space underground<br>-not aware of one's health problems<br>-lack of PPE	-Source of livelihood<br>-bought personal detectors and formed a company<br>-acquired land, cars<br>-managed to pay school fees for kids.<br>- No known health impacts	-Encouraging use of complete PPE<br>-Exploring why people are not using PPE<br>-Education
**18.**helping people, procurement, amalgamation (sponsor)<br>**Male 39**	PPE	-Use of acid during amalgamation<br>-amalgam burning<br>-dust<br>-fumes from explosives	-helmets<br>-work suits<br>-gumboots<br>-safety shoes	Yes<br><br>Every time	-ignorance<br>-lack of value for PPE	-Livelihood source<br>-buying cattle<br>-building a homestead<br>-paying school fees<br>Worries on the long-term impacts of ASGM	-PPE<br>-Education
**19.** Mining after blasting<br>Amalgamation<br>Burning amalgam<br>**Male 24**	PPE; gloves, helmets, work suits<br>Gumboots<br>Dust mask<br>Respirator	Mercury from amalgam burning<br>Going down by a bucket (winch)	Work suit	Yes<br>Every time when working	Water flowing and dripping	Managed to acquire cattle, goats, phone<br>-No known health impacts	-Replacing winch and rope with head gear<br>-wheelbarrow<br>-complete PPE
**20.**Migrant miner who follows gold rushes and travels all over the country.<br>Mining<br>Crushing<br>Amalgamation<br>Amalgam burning<br>Accidents were common in gold rushes. The people help each other in case of accidents. Clinics and transport is usually available. The injured raises money for hospital cost.<br>**Male 32**	PPE: helmets gloves respirators (people in small-scale mining not used to respirators)	-Too many people in an old shaft 5000 or more people working with no control;<br>-Uncontrolled blasting<br>-weak points and rock falls 48 or more levels<br>-	Work suits<br>Gumboots<br>Helmets	Yes Consistentl y	Informal miners don't get money all the time	-Livelihood source; school fees for children, acquiring property<br>-acquiring mining equipment<br>-buying inputs (pump) for farming(gardenin g)<br>-building a homestead<br>-Long-term health impacts from mercury and dust<br>Most people have TB	-Medical check-ups like medical clinics from New Start Centre<br>-Educating people on medical check ups<br>-mine owners should provide PPE<br>-Enforcement of use of PPE and safety measures which includes firing non-compliant workers<br>- Miners could also be required to own PPE before recruitment
**21.-** Fitting compressors and pumps for the miners (can go down by rope to fix	PPE:<br>gloves,<br>helmet<br>work suits<br>goggles	Dust<br>Going down by rope	Work suit<br>Helmet<br>Glasses<br>Gloves	Yes<br>Always	-Heat underground<br>-money to buy masks	More money from miners: livelihood source, supporting family	PPE provided by mine owner

water pumps)<br>-fixing stamp mills<br>**Male 60**	gumboots					-Unknown health impacts	
**22.** Mining,<br>Dry drilling<br>Crushing<br><br>**Male**<br>33	PPE;<br>Helmet<br>Work suit<br>Gumboots<br>Respirators<br>Dust mask	-Falling rocks<br>-Mercury is difficult to determine without health check-ups	Gumboots<br>work suits	Yes<br>Consistentl y	Insufficient income, working to meet family needs	-More paying than agriculture. Managed to get married, raise a family pay fees for kids<br>Bought cattle<br>-Unknown health impacts	-Complete PPE<br>-Site owner's to discuss with workers on advancing money for PPE
**23.**Mine owner<br>Delegation and supervision of mining, the winch and rope was used for going down<br>Amalgamation<br>Amalgam burning<br>Safety first, use of PPE<br><br>**Male 28**	Mask<br>Muslin cloth	-Carbon from previous mining activities<br>-unstable rocks<br>-mercury: people love money, don't realize the dangers but it's dangerous	Work suits<br>Helmets<br>Gumboots<br>Touches	Exactly	Money<br>education	-Driving a Lexus<br>-Owning a house in low density<br>-paying school fees for kids<br>-could afford to drink beer<br>- Health impacts, 'health still ok'<br>-'health check-ups, a problem. Serious problem with money	-Equipment for mining<br>-good mining techniques<br>-PPE<br>-Safety first
**24.**Manager **male 45**<br><br>Get the team to work:<br>safe precautions<br>the right attitude<br>clearing the work place<br>mediation<br>making sure everyone is safe<br>Commented that some injuries were from carelessness<br><br>Availability of daily needs: work and social	-Open mines for former informal miners to learn new things on PPE and safety<br>-resources when projects are at infant stage (limited resources, limited safety gear)<br>-diet is affected by resources	-Condition of the mine, need for strong shaft and roofs to avoid rock falls<br>-Gases, greater carbon in some points<br>- Noise was not yet a big issue<br>-Mercury was described as highly poisonous generally people do not use gloves when handling mercury	-Workers use their own, mine still at development stage.<br>- Work suits will be provided at commercial stage. Only helmets were provided at development stage	Yes<br>High prevalence of injuries without PPE	-heat<br>-not getting used<br>-Misguided thought for workers who believe that they cannot wear PPE<br>-lack of education<br>-people prioritizing other things and not PPE<br>-people resist PPE because of limited resources<br>-Underground-Safety kit works with resources<br>- The longer the development stage the longer the period of limited resources<br>-Workers with informal mining background who are used to working without rules find it difficult to embrace rules<br>-negative attitudes ignorance	-Being away from home and family<br>-Can be indefinite, not affording visit home, there is no servant quarters men live together in temporary shelters<br>-Long term impacts on health: TB, lack and improper use of PPE	-government formalizing more formal ways of working with standards and rules<br>- need for financial support<br>-setting a window period to formalize things (including paying taxes) and meet safety(requiremen ts)<br>-People forced to comply
**25.**Underground mining, dry blasting, 4 hours waiting time after blasting<br>Making the environment safe,	-respirators because of dry drilling	*Dust<br>-jag hammer very noisy<br>-accidents not common	Work suits<br><br>Gumboots<br>helmet<br>gloves	Yes<br>Every time	-The mine supplies PPE but people are not used<br>-Very hot but one can still work with	TB is common in Small-Scale mining	-PPE to reduce degree of injuries<br>-Firing non-compliance as in Large mines

bringing down lose rocks<br>**Male 43**					PPE		
**26.**Mining after dry blasting<br>Lashing after blasting<br>Going down by manual winch<br>**Male 27**	PPE	Carbon gases after drilling	Helmet<br>Gumboots<br>Dust mask	Yes<br>Every time	-Type of the pits could be hot. PPE is good to prevent exposure to dust, accidents, noise and mercury	-Livelihood source: money to take care of the family<br>-Acquiring wealth and property<br>-Long-term effects without protective clothing	-Safe working environment including fencing<br>-PPE<br>-Regulations for small-scale miners to avoid risks and accidents
**27.** Lashing after dry blasting<br><br>**Male 19**	Safety Protective clothing	Death<br>Rock falls<br>Pits not strong	No protective clothing	Yes<br>Sometimes	Not known	Not answered	PPE
**28**.Supervising workers who are developing a mine<br><br>**Male 30**	Milk for the workers to clear dust after blasting, mining and lashing<br>PPE: helmets, worksuits, safety shoes, safety chain, waist belts	-Minor injuries because of no compliance to consistent use of PPE<br>-Carbon from previous mining activities(old mine)<br>-*Head shaft to replace the manual winch<br>-mining equipment(generators, pumps)<br>NB*Expressed that mercury is not as poisonous	Respirators work suits	Yes<br>Consistent use	-mining employees who resist consistent use of PPE.<br>1 miner who got minor injuries from a rock fall due to inconsistent use of PPE was fired<br>-new miners lack experience and think temperatures underground are too high to work with PPE<br>-New miners not interested in using PPE	-gaining wealth, happiness and joy<br>-unknown health impacts	-training<br>-engaging medical doctors and nurses in ASM &health<br>-Consistent use of PPE must be signed for as part of the recruitment contract<br>-mine owners must provide PPE<br>-Informal miners should be trained and monitored on consistent use of PPE by the association of ASM<br>-Implementation of the occupational and safety regulations used in large mines in ASM
**29.**mining after blasting<br><br>**Male 28**	PPE	-dust<br>-compromising waiting time to mine after blasting	-safety shoes<br>-helmets<br>-gloves<br>-respirators	Yes	Number of pairs. Was not wearing work suit because he had 1 pair which had gone for laundry	-Bread winning<br>-health impacts not known	-tablets for miners for common ailments<br>-chemicals to treat drinking water<br>-enough pairs of PPE
**30.** Mining gold and iron **Male 55**<br><br>Had experienced injuries because of non-use of PPE because of high temperatures underground. The mine had incident of injury from a rock fall. The injured employee had no PPE and was fired	-Milk for clearing dust after exposure<br>-PPE: helmets, hard hats, work suits,<br>Safety belt<br>Safety chain	-Carbon from previous work<br>*Head shaft to replace the winch<br>-Lack of generators and water pump<br>-Lack of -cyrene to warn people of noise before blasting<br>-Dust	-respirators<br>-more work suits	Yes<br><br>Every time	-Inexperienced new minor not using PPE underground due to high temperature's<br>-Lack of interest in PPE	-Economic benefits which resulted in wealth, happiness and joy.<br>-Unknown health impacts	-Training on safety and PPE<br>-Engaging medical doctors and nurses<br>-Mine owners should provide PPE<br>-Compliance to PPE should be included in the employment contract<br>- Small scale miners should be trained under the association of ASM (ZASMA)<br>-The regulations on mine safety

							should be implemented
**31.** Underpaid, underground mining and driving<br><br>**Male 44**	Safety is lacking	Many dangers, lack of PPE underground, dust,*chemicals at cyanidation, noise mercury	Work suit only	Yes<br>Every time	-Personal views on self-protection<br>-reluctance and laziness<br>-no routine check-up less awareness on the impact of exposure to health risks in ASGM	Long term impacts due to exposure to dust, injuries and chemicals resulting in health impacts such as TB	-Mining equipment<br>-PPE
**32.** Mining<br>Cyanidation<br>Lashing<br>Panning/concentration/ amalgamation<br>Amalgam burning.<br>Common issues were accidents and falling pits<br><br>**Male**<br>**34**	-PPE<br>-Milk and maheu (local liquid food) to clear fumes and chemicals from blasting	-Infections (not specified after probing)<br>-Dry blasting<br>-Injuries<br>-Rock falls<br>-Mercury blowing by mouth which was later replaced y barrel<br>-Thought there was no risk with mercury because he had not heard of anyone affected	Worksuits<br>Gumboots<br>Helmets<br>Gloves<br>Touches	Yes	-People not using PPE sometimes when they are not feeling fine	-Ignoring symptoms of sickness	-PPE-work suits, touches, helmets<br>-food
**33.** Mining after blasting. Blasting could be wet or dry. Blasting was followed by blowing of gases by compressor. Workers could re-enter when fumes were still there<br><br>**Male**<br>24	-drinking maheu or beer after blasting<br>-Supervisors should check safety before re-entry of workers	Injuries (lack of use of PPE)	No PPE, sponsors were not providing	Yes<br>All the time	Could not think of any after probing	-Injuries,<br>-risk of collapsing mine<br>-risk of death<br>-can spend 24 hour in the shaft<br>-many people working underground<br>-exposed to underground water	Could not think of any after probing
**34.** Undergrounding mining<br>After blasting<br>Wet and dry blasting<br><br>**Male 27**	-safety starting from home to work<br>-PPE<br>-Touch with power<br>-lose rocks	-falling rocks<br>-dusty surface<br>-wet conditions underground<br>-*carbon from blasting	-gumboots<br>-work suit<br>-helmet<br>(Was wearing the PPE)	Yes<br><br>Consistently	- Need for people to accept health risks in ASM	small money<br>-unknown long-term health impacts	Regulations: ASM workers must not be allowed to work without PPE
**35.** Assistant manager Cyanidation **Male 26**<br><br>He was concerned about poor safety and workers who come to work drunk while they work with cyanide<br>Anti-dote was admistred as first aid for accidental cynide exposure	-Wearing PPE(gumboots, respirators, good gloves)<br>-Anti-dote for First Aid<br>-Alertness when handling dangerous chemicals<br>-Drinking milk after duty	Chemicals	Respirators<br>Gumboots<br>Gloves	Yes<br><br>Consistently	-Difficult to wear PPE in summer season because of high temperature<br>-Cannot spend the whole day wearing a respirator because of difficulties with breathing	-Not safe to work over 5 years because of side effects from chemicals (asthma)<br>-shorter life	-Education<br>-Organizations that help ASM miners
**36.** Gold buying	-Safety first,	-Haphazard blasting	Few items of	Yes	-Lack of complete	-People can	-Advice

mining<br>Mine manager<br>The current mine was 32m deep. Injuries were few because of proper safety measures<br><br>**Male 34**	PPE<br>-Sanitation: safe drinking water and toilets (including underground)	-Blaster must be followed by timber men after the clearing of gases to check safety before –lashing<br>-noise<br>*-dust<br>-mercury	each, (not free to give more information)	Every time	set of PPE<br>-Pride and love of money despite risky working conditions	make wise or unwise decisions with money<br>-dust<br>-injuries<br>-mercury intoxication	-Education<br>-Informal miners should buy their own PPE
**37.** Mining after wet blasting<br>Amalgamation<br>Amalgam burning<br>Collapsing mines<br><br>**Male 33**	Prevention from carbon from blasting and mercury poisoning	*Carbon from previous mining activities<br>-collapsing mines<br>-dust	Worksuit<br>Gumboots<br>helmets	Yes<br>Consistently	-Workers not used to using PPE<br>-Lack of understanding<br>-Missing PPE items, not provided by mine owner<br>-Limited resources<br>-Not caring: " When your time has come it (accidents) can still happen" (despite wearing complete PPE)	-The money is not consistent, it only pays when you are lucky.<br>-Long term health effects of dust	-Financial backing for small-scale miners<br>-Compete PPE<br>-Education<br>Complained of empty promises of financial assistance
**38.**Mining: no blasting<br>Amalgamation<br>Amalgam burning<br>**Male 20**	Unknown health needs at work	Bad working conditions	Gumboots<br>work suits	Yes	Lack	Dust<br>Poor hygiene	PPE
**39.**mining<br>Cyanidation<br>Amalgamation and amalgam burning<br>**Male 48**	Safety	*cyanide<br>Mercury<br>Acid<br>Caustic soda	Safety shoes<br>Worksuits<br>gloves	Yes<br><br>'Everything every time'	-Not accepting the need for PPE<br>-Despising PPE	Drinking dirty water with carbon when working underground	Underground manager
**40** Mining after wet blasting<br>There were concerns on injuries from rock falls and lose underground old mines. Injuries were happening once or twice a year. Cases of injuries were reported to the police and the general hospital<br>**Male 27**	PPE	-rock falls<br>-noise and machinery<br>*Mercury, the impact is chronic and people use it without protection.<br>-Dust from dumps<br>*Cyanide, harms without knowledge, might not be avoidable like injuries.	Helmet<br>Gumboots<br>Worksuits	Yes<br><br>Consistently	-Lack of knowledge<br>- Realizing the importance late<br>-Ignoring somethings like masks and focus on PPE associated with immediate impacts like safety shoes	No health problems from mining, mining is a livelihood source, if you don't mine you get nothing	-Education on masks<br>-Strict regulations, mine workers must not be allowed to work without PPE.<br>-Sponsors should provide PPE
**Describe your usual working day**	**What are your health needs at work**	**Describe what could be dangerous to your health at your workplace**	**Which PPE do you have at your workplace**	**Would you use PPE if you had a choice**	**What are the challenges with using PPE**	**What is the impact of mining on your health and your daily life**	**Possible health interventions**
**41.** Mining after wet blasting.<br>Injuries from mine collapse in the rain season<br>Cases were reported<br>Injured workers were taken to the	-Gloves<br>-Eye protector	*mine collapse<br>-dust<br>mercury	Helmets<br>Milk and local liquid food maheu	Yes<br><br>Every time	Lack of PPE	-Not much, bought 2 cattle<br>-Possible health impacts chest problems from exposure to carbon	-PPE<br>-Mining equipment

hospital<br>**Male 33**							
<u>42</u> Underground mining<br>**Male 58**	Ok	Water<br>Lack of PPE	Helmet<br>Gumboots<br>Safety shoes	Yes	Not difficult	No impacts	PPE<br>Food<br>Good pay
**<u>43.</u>**Drilling<br>Wet blasting<br>Incomplete safety measures and recklessness was described as a source of injuries<br>**Male 29**	- PPE<br>-Drinking milk, chibuku (opaque beer)	-Carrying explosives<br>-Accidents	Gloves<br>Helmet<br>Glasses<br>Earplugs<br>Respirator<br>Raincoat	- Yes<br><br>Practicing secondary (formal) mining	Wet conditions<br>-Reluctance<br>- High temperatures<br>- poor ventilation	- Source of income to acquire mining equipment<br>- Chest problems caused by drilling and blasting	- Mining equipment<br>-Mine owner should provide PPE<br>-Informal miners should buy their PPE
**<u>44.</u>** Lashing after wet blasting<br>Blasting was followed by blowing with compressor<br>Lashing was done 4 hour after blasting<br>Rock falls took place once in a while<br>**Male 32**	PPE; helmet<br>Milk for drinking after lashing	*gases from blasting<br>-dust<br>-injuries	Gumboots<br>Helmets<br>Gloves<br>Respirator(no dust mask)	Yes<br><br>Everday by law at the mine inspection done when going down	No challenges, it is for my own health	Drinking contaminated water (open defaecation &urination) underground	The participant was a TB patient on 6 months medical leave. The mine had paid for his hospital fee. He needed money for food since he was on strong TB medicine which requires nutritious food
**<u>45.</u>**Blasting with protective clothing. Drinking soup after blasting. Had experienced rock cuts and minor injuries<br>**Male 24**	Respirator	Dust(wet blasting)<br>Injuries<br>Noise (had no earplugs)<br>'Mercury helps get the gold and there is nothing not dangerous'	Gloves<br>Helmets<br>Gumboots<br>Work suits<br>Eye protector	Yes	Ear plugs block hearing	-TB despite using respirators<br>- Many cases of fractures and at times death	-PPE
**<u>46</u>** Lashing after dry blasting. Shaking during blasting causes cracks, falling rocks and collapsing mines which can cuase fatal injuries beyond rescue. Fatal injuries were common once in a while<br><br>**Male 25**	Respirator<br>-Maheu, opaque beer to drink after duty to clear gases	Dust<br>*collapsing mines<br>-going down by rope in a bucket<br>-Long term impacts of mercury on eye-sight	Gloves<br>Helmets<br>Gumboots<br>Work suits<br>Eye protector	Must<br><br>Always	Ear plugs block hearing during communication<br>-Goggles can fall down when loading	-Livelihood source<br>-Potential to buy property. Participant had bought 4 cattle and was managing to raise his family.	Using PPE underground<br>-Capital<br>- Social Security insurance<br>- Educating workers
<u>47</u> Amalgamation<br>Lashing after dry blasting<br>Drinking milk/maheu after duty<br><br>**Male 24**	PPE: respirators	<u>Surface</u><br>-Injuries from low PPE use<br>-invisible effects of mercury<br>-noise<br><u>Underground</u><br>*rock falls<br>*carbon fumes from blasting	Helmets<br>Earplugs<br>Work suits<br>Gloves<br>gumboots	Yes<br><br>Always	-Depends on where and one started mining, one can get used if PPE was in the system in the first job<br>- It is difficult to breath with respirators	-Gases<br>-falls<br>-lack of PPE when working	-education<br>-enforcing regulations on PPE, fine and firing
**<u>48.</u>**Lashing after dry blasting<br>**Male 31**<br><br>Watering done	Wet blasting	*Carbon fumes after blasting<br>Mercury not very dangerous and not very safe	-All PPE from mine owner<br>-replenish : work suits after 6	YES	-Some mine owners are not willing to buy for the workers.<br>- Lack of strict	The health impact is reduced with PPE	Sourcing money for PPE

before lashing .<br>Safety procedures at his work:<br>-Read safety safety when getting to the mine<br>-Told safety first in Shona<br><br>-Firing was the penalty for non-compliance with PPE and (no one had been fired)<br>-Each had 1 pair of work suit and people were managing to wash and bring the work suits to work everyday			weeks(sic)<br>Safety shoes after 3 months<br>Respirator after 6 months		management<br>-Gumboots must be used with a powder to avoid heat blasters<br>- The jag hammer should be balanced to make it possible to use gloves<br>-Cooling fan needed underground to reduce temperatures<br>-Standard –size tunnels		
**49** Management<br>**Male 37**	-Favorable working conditions<br>-mining equipment<br>-dry drilling<br>-mining ventilation	Rock falls	Worksuits<br>Gumboots<br>Respirators<br>earplugs	Yes	-Workers not putting on PPE especially raincoats<br>-Workers losing their PPE<br>-Workers not understanding the importance even after paying penalty	-Poor ventilation can cause diseases such as TB. TB is very common. Workers with TB were encouraged to seek medical attention (mine owner covering hospital expenses) and avoid spreading TB	-Education on the importance of PPE<br>-Enabling miners to have medical AID cover<br>-Improve mine ventilation
**50.**Lashing, 3 hours after dry blasting<br><br>**Male 25**	PPE:<br>helmet, work suits	Falling rocks<br>(Not aware of impacts of carbon gases and falling rocks)	Gumboots only	Yes<br>Consistentl y to reduce accidents	-Lack of PPE<br>"Everyone wants PPE"<br>-"People are different, some people are committed to using PPE"	Can cause injuries<br>"Mines are different, some mine managers check the mine after blasting"	Safety and more care from mine owners
**51.**Feeder at the milling plant Amalgamation and cynadation at times. Had not exoerienced accidents from cynadation<br><br>**Male 36**	"Nothing"	The milling plant<br>*mercury (panning and burning amalgam)<br>Noise without earplugs<br>Dust without dust mask	Helmet<br>Gumboots<br>Worksuits<br>Gloves<br>Earplugs<br>Respirator	Yes<br>Sometimes	-Cannot use gloves when working with grease<br>-Some workers don't use PPE because of stubbornness (recommended charging 20 USD for non-compliance with PPE)	Without PPE, poor health due to exposure to cyanide, mercury, dust and milling	-Mine owner paying for regular medical check ups<br>- Charging staff on non-compliance to PPE
**52.**wet blasting without explosives. Injuries from small rock falls. Concerntration<br><br>**Male 29**	'everything is good'	*falling rocks<br>-mercury is dangerous	-Gloves<br>-Helmets<br>-Work suits<br>-Gumboots<br>-no masks<br>No earplugs	Yes for the relevant PPE.<br>Mask was described as not relevant because there was no dust and the participant was not involved in	Not used to ear plugs	Mining is not healthy<br>Can result in death if not safe	-Inspection of shafts and consistent use of PPE<br>-Unsafe shafts must be closed

				amalgam burning			
**53.** Drilling<br>Lashing after dry blasting<br><br>**Male 28**	PPE	Injuries<br>Collapsing mines	Helmets<br>Gumboots<br>Work suits<br>Earplugs<br>Respirators<br>Sometimes gloves	Yes<br><br>Regulations	Non-compliance, some people compromise when the manager is away	Depending on what you do with the money, like negligence on mine development.<br><br>No responses to health impacts	Security to avoid robbery<br>PPE
**54**. Director of emergencies<br>Amalgamation<br>**male 44**<br><br>Amlgam burning. There was an experienced mine manager and blaster. Blasting was done while researchers were collecting data. Blasting was followed by blowing by the generator. It was reported that after 4 hours the professional manager and the driller check if safe before lashing. PPE was supplied by the mine. Replacement PPE was provided through exchange with old PPE	Concerned about health for workers especially raincoats to prevent workers from underground cold and pneumonia<br>PPE	Enough air underground (gases.<br>-PPE<br>-safe electricity underground<br>-Safety first, Ema requires visible signs<br>-dry drilling<br>-using the retort for mercury burning<br>-<br>-injuries(avoided by safe talks	-Helmets<br>-Worksuits<br>-Raincoats<br>-touches<br>-ear plugs(not enough for everyone exposed)<br>No gloves masks(both dust masks and respirators	Yes<br><br>Management charged penalty for non-compliance to PPE regulation	-Losing PPE, some people join the mine as employees and stop coming after a day or so after receiving PPE<br>- If workers receive 2 pairs of PPE they sell the second pair	-Know what is needed and do it very well, you will cope with risks<br>-If you don't know and you don't do it right workers will get sick at any time	-Continual learning Zimbabwe School of Mines<br>-Monitoring workers when they are working, 'workers who were previous informal miners want to mine according to their former mining habits in artisanal mining
**55.**Manager. Daily routine: prayer, safety talks and delegation<br><br>**Male 62**	PPE<br>Checking equipment<br>5 points of safety	*"After blasting wait for 4 hours before re-entry, gas is the most dangerous thing in the mine"<br>-Dust with dry drilling<br>-Injuries<br>-noise<br>- "Some people say mercury is dangerous but I don't know"	Helmets<br>Light<br>Gumboots<br>Raincoat<br>earplugs	Yes	Dust mask blocks and makes it difficult to breath<br>Lack of safety talks	Bad working environment	-Programmes on strict implementation of safety regulations
**56.** Drilling, blasting and lashing.<br>Concern was expressed on safety<br><br>**Male 49**	-Health working environment with no gases<br>-PPE	Coming to work drunk<br>Poor communication<br>Gases and dust	Gumboots<br>Worksuit<br>Helmet	Yes	Difficult to replace especially the small items of PPE	Lifting heavy things can cause back pain<br>Dust<br>Gases	Health working environment<br>Good communication<br>Miners' association to help with PPE
**57** Dry and wet drilling<br><br>**Male 53**	PPE: protection against dust	Dry drilling<br>Unsafe shafts<br>Working in the shaft after blasting, before 4 hours	Dust mask<br>Earplug<br>Helmets<br>Worksuits<br>Gumboots	YES	Dust mask cannot work when damp	Overworking<br>Staying long in hard water<br>Participant was not free to talk about dust and TB	Regulations to stop dry drilling.<br>Healthy working environment: toilets and safe sources of drinking water
**58.** Lashing<br>Assisting in all areas<br><br>**Male 22**	Food that protects the body	*TB and dust<br>Noise<br>Injuries were minor	Gloves<br>Mask/mutton	Sometimes	Some people are more comfortable with working in shorts	Back ache<br>Not aware of impacts of dust and carbon	Milk, maheu and soups for dust

**59.**Was new at work. Surface mining<br><br>**Male 31**	PPE	Not yet aware	Helmet<br>Worksuits<br>Gumboots<br>Raincoats	Yes<br>Every time	Not aware	Not aware	Not aware
**60.**New at work. Mining<br>**Male 30**	PPE	Fumes from blasting	Had not yet received	Yes<br>Always	Not aware	Not aware	Being smart
**61**.Lashing, 4 hours after wet blasting<br>-accidents were not common, workers were working cautiously<br>**Male 28**	PPE :gloves, masks	*lose rocks after blasting<br>*gases after blasting<br>No noise<br>No dust<br>No amalgamation	Helmets<br>Worksuits<br>Gumboots<br>No gloves and respirators<br>No need for masks and ear plugs	Yes<br><br>Every time	-Workers must buy gloves and respirators, they are not available at work<br>-some people don't priorities PPE<br>- not a requirement at work	-Source of livelihood<br>-breathing gases which affects the chest	-Working with PPE<br>-Educating informal miners
**62**. Lashing 4 hours after wet blasting. Mine blaster inspects before lashing which avoids injuries<br><br>**Male 30**	PPE: raincoats	No dangers: shaft is alright, no dust (too much water), no injuries,	Helmets<br>raincoats<br>Worksuits<br>Gumboots<br>gloves and<br>No need for respirators, dust masks and ear plugs	Yes<br><br>Every time in every shift	No challenges: always reporting challenges to management<br>-Mine provides PPE	Acquiring property, paying school fees for kids.<br>-No health impacts: good air underground, no dust, no water no known health hazards	-Use of complete PPE<br>-help informal miners according to how they work
**63**.Drilling using chisel and hammer, no blasting. Had experienced minor injuries. Amalgamation<br><br>**Male 26**	Everything is provided	no dangers everything is correct'<br>Dust	Helmet<br>Worksuits<br>Gumboots<br>Gloves<br>Touch<br><br>No respirators, dust masks and ear plugs (was using earphones)	Sometimes 'I can go underground like this' with no PPE	I have 1 pair which is always dirty I leave it underground. I don't wash my work suit.<br>'This is my work suit also' (his clothes)<br>-'everything is proper'	Everything is correct. Got medical examination before induction	Just tell them work first
**64.**dry drilling Amalgamation, amalgam burning. Managers' survey the ground and stop workers if conditions are not good.<br><br>**Male 25**	PPE	Dust (no mask)<br>Injuries can happen any time because of tiredness)<br>-noise when drilling, no ear plugs.<br>Mercury is source of money, harmful only when swallowed	Helmet<br>Gumboots<br>Worksuits (provided by company on the first day of work)<br>No gloves, masks, goggles and earplugs)	Use<br><br>Everyday	Small items, not available at the mine or milling center	Bread winner for siblings<br>Long term health effects	Teaching and discussing<br><br>Informal miners must buy for themselves
**65**. Mining4-5 hours after wet blasting in an inclined shaft. Injuries not so common. Use of PPE reduces injuries. Overseer miner makes routine checks of safety before mining<br><br>**Male 25**	Health needs are taken care of by the mine	Rock falls<br>Not wearing PPE<br>*Gases from blasting	Gloves<br>Helmet<br>gumboots<br>earplugs<br>safety belts	Yes<br><br>Every time	No problem. The problem is when you don't wear	Not known	-Respecting the waiting time after blasting<br>-checking safety before going down
**66**. Mine owner<br>**male 37**	PPE: masks Maheu and	The shaft should be well-timbered	Helmets<br>Gumboots	Yes<br>All the time	Some people don't use because	Client was not free to discuss	Encourage use of PPE

Checking what is needed for the day.<br>Checking safety in the shaft, safe first<br>Amalgamation<br>Amalgam burning<br>Had a case of accidents 2 years back when 2 guys entered the mine before checking, the mine collapsed and the guys were closed in(participant broke down and could not give further details about the incident)	milk were provided to drink after work		touches		they are not used	health impacts	
**67**. Participant had no time for qualitative questions<br>**Male 28**							
**68**.Mining after dry blasting, waiting time 2 &1/2-3 hours<br>Company provides PPE<br>**Male 34**	Respirators<br>Air link for drilling<br>Accidents were not common	-Safe drinking water in the mine in general and underground.<br>*dust<br>-noise<br>-fumes from blasting<br>*mercury is very dangerous	Helmets<br>Gumboots<br>work suits	Yes<br><br>Every time	-ignorance<br>-challenges with provision of small items of PPE which was described as lack of care for the health of the workers	-Manual machinery and vibration can affect the chest<br>-Long term effects of TB<br>-Carbon is fatal	
**69**.Gold buying<br>Amalgamation<br>Amalgam burning<br>Clint spent much of his time at the mill center. Common accidents were loss of fingers when collecting the sands (milled ore) from the box. The stamps can move by mistake due to miscommunication with the mill operator and cut off the fingers<br>**Male 48**	-Avoiding drinking beer at the mil<br>-good communicati on	*losing fingers<br>-cyanidation<br>-mercury	Helmets<br>Safety shoes	Yes<br>Sometimes	-People not used to PPE.<br>-People use mercury without protection despite knowledge of mercury contamination, people like money	Long term impacts of mercury even to the people around you	-Encouraging use of PPE<br>-Limited resources some people want to buy PPE but don't have resources
**70.** Buying ore<br>Clear ore and milling<br>Mixing cyanide and surf and panning<br>Amalgamation burning<br><br>**Female 31**	Safety requirements<br>:<br>complete PPE<br>- -amalgam burning	Don't sit close to the shaft, stay 50m away(fined 50USDfor staying close the shaft<br>- Don't make the bucket too full(too heavy for the back)	Helmet<br>Raincoat<br>Work suit<br>Safety shoe/gumboo ts (buy for self)	Yes<br><br>work suit always	Protection is good, you can identify each other<br>-Was not aware of the importance of the mask	Changed my life and children<br>-Earning a living<br>-Drinking maheu and milk to protect my health	-Enough food and drinking<br>-Maheu
**71.** Buying ore<br>Clear ore and milling<br>Mixing cyanide and surf and panning<br>Amalgamation burning	PPE	Milling<br>dust	work suit<br>Safety shoe<br>helmet	Yes<br><br>Sometimes	Hot weather<br>Respirators too tight	-Much changes; I can pay school fees for the kids<br>Bought a car<br>Bought a stand<br>Finished building a house	Enough supplies of medicine at the local clinic<br>HIV& AIDS tests and treatment

**Female 48**						Supporting the family No health impacts. I take antibiotics sometimes	
**72.** Buying ore<br>Loading ore<br>Clearing ore<br>milling<br>panning<br>Amalgamation<br>Amalgam burning<br>**Female 44**	PPE: work suit, helmet, safety shoes	Accidents, falling ore<br>dust	work suit<br>Safety shoe<br>Helmet	Yes<br><br>Sometimes	Hot weather	-Much changes; I can pay school fees for the kids<br>Bought a stand<br>Built a house(in Harare)<br>Supporting the family orphans<br>bought a smart phone<br>unknown health impacts.	Medicines<br>HIV & AIDS treatment and test at the local clinic<br>Capital to buy ore
**73.** Buying ore<br>Loading ore<br>Amalgamation<br>Amalgam burning<br>**Female 43**	PPE gloves, respirator medication	Dust not an issue<br>Not aware of the impacts of mercury, thought it was surf to burn while standing several meters away	work suit<br>Safety shoe<br>helmet	Yes<br><br>Sometimes	Hot weather<br>Failure to get safety shoe size, size 6<br>- Challenges with helmets when carrying buckets<br>- Helmets get missing if left on the ground	-Much changes; I can pay school fees for the kids<br>Paying hospital bills<br>Able to support myself, no longer depending on men.	Not answered
**74.** Buying ore and milling<br>**Female 36**	PPE: dust mask, respirators	Dust<br>Mercury, (we need mercury, no mercury no work)	work suit<br>Safety shoe<br>Helmet	Yes<br><br>Always(was not wearing helmet and safety shoes)	Hot weather<br>Difficult to carry buckets with helmets<br>"I like work suits even in hot weather, it covers the body)	-Good changes; I can pay school fees for the kids (husband had moved out)<br>Bought property<br>Caring for her mother<br>Better life<br>Can afford to eat health food	
**75.** Buying ore<br>Clear ore<br>Loading to get ore<br>Amalgamation<br>Amalgam burning<br>**Female 35**	PPE: work suits, helmets, gloves	Dust at times	work suit<br>Safety shoe<br>helmet	Yes<br><br>Sometimes	Hot weather<br>Gumboots and safety shoes too heavy<br>Not used	- I can pay school fees for the kids<br>Bought a car<br>Moved town and managed to rent<br>Bought a stand<br>Now building a house<br>Supporting the family No health impacts<br>No health impacts.	-Medical check ups<br>-Medication including ARVs<br>-Family planning tablets<br>-Upgrading the local clinic to a hospital
**76.** Loading to get ore<br>Amalgamation<br>Amalgam burning<br>**Female 38**	PPE: gumboots, work suits, helmet, respirator, dust mask	Minor accidents when not wearing gloves<br>Dust<br>Not aware of health impacts from mercury	work suit<br>Safety shoe<br>Helmet	Yes<br><br>Sometimes (work suit all times)	Not possible to carry buckets with helmets	-Very much No stress on getting fees for the kids<br>Bought property	PPE; dust mask, respirator
**77.** Buying ore<br>Clearing remaining ore<br>**Female 37**	PPE (gumboots, gloves, respirator) antidote	mercury<br>dust<br>*cyanide	work suit<br>Safety shoe<br>Helmet	Yes<br><br>Sometimes	Hot weather<br>Gumboots are heavy	-It's good family has improved<br>Long term impacts of dust<br>.	Respirator<br>Antidote<br>Dust mask
**78.** Buying ore<br>Amalgamation<br>Amalgam burning<br><br>**Female 34**	Safety shoes<br>Helmets<br>Work suites	Cyanide<br>mercury<br>dust	Work suits<br>Safety shoe<br>Helmet	Yes<br><br>Sometimes must at the mill	Hot weather	-Much changes; I can pay school fees for the kids<br>Bought a car<br>Bought a stand	

						Finished building a house<br>Supporting the family No health impacts.	
**79.** Buying ore<br>Clearing remaining ore<br>Amalgamation<br>Amalgam burning<br><br>**Female 37**	PPE: work suit	Dust<br>Did not know impacts of mercury	work suit<br>Safety shoe<br>Helmet	Yes<br><br>Sometimes	Hot weather<br>Knowledge and availability<br>Gloves get wet when panning	-Very much changes; I can pay school fees for the kids<br>Bought a stand<br>Buying food for the family<br>No stress No stress better health.	No suggestion
**80.** Health Department participant<br>**Female 53**	PPE<br>Health food<br><br>Clean water	-Checking the bucket<br>Should not carry many things when carrying the bucket<br>Keeping toilets clean<br>Avoiding diarrhea<br>DUST	N/A	N/A	No challenge regulations for mining must be followed<br>Attitudes inexperience	Not answered	No suggestion
**81.** SAFETY Department<br>Explaining safety regulations and standards in ASM<br>Shaft and equipment inspection<br>Education on keeping a health environment<br>**Male 36**	NGO's coming for medical check-ups<br>Working on funeral policy<br>Mine owners take care of medical costs<br>NSSA teaches on accidents	* Spinal injury<br>Cyanide and mercury<br>Chemical contaminated water<br>*dust	work suit<br>Safety shoe<br>Helmet<br>Goggles<br>Gloves<br>Earplugs<br>Dust masks	Yes	Workers not used<br>lack of inspection	-back ache<br>open fractures<br>more danger<br>dislocation<br>slipping<br>falling<br><br>.	Compliance to regulations on safety<br>Training
**82.** Buying ore<br>Amalgamation<br>amalgam burning<br><br>**Female 31**	PPE: Mask, gloves	mercury<br>Dust<br>Accidents not so much for women	work suit<br>Safety shoe<br>Helmet	Yes<br><br>Sometimes	Hot weather	-Husband not working<br>Help with paying fees<br>Buying enough food and clothes<br>.	Education on dust, carrying heavy things and mercury
**83.** Health Department<br>Attend minor injuries, cuts and STIs<br>**Female 24**	PPE safety shoes work suits<br>Safe source of water	Dust<br>STIs<br>Low use of PPE	N/A	N/A	Financial<br>hot weather	Disrupted family life, unprotected sex STIs<br>Dust<br>Medical equipment	Better health facility<br>Safe source of water<br>Workshops on safety and health registration<br>drugs<br>refrigerators
**Describe your usual working day**	**What are your health needs at work**	**Describe what could be dangerous to your health at your workplace**	**Which PPE do you have at your workplace**	**Would you use PPE if you had a choice**	**What are the challenges with using PPE**	**What is the impact of mining on your health and your daily life**	**Possible health interventions**
**84.** Sponsoring mines<br>Amalgamation<br>Amalgam burning<br>**Female 43**	PPE helmet work suit	Mercury<br>dust	work suit<br>Safety shoe<br>helmet	Yes<br><br>Sometimes	Hot weather	I can pay school fees for the kids<br>Not lacking food at home<br>Phones for kids<br>.	PPE work suits, gumboots, helmets, mutton cloth, masks

